# 5-Fluorouracil sensitizes colorectal tumor cells towards double stranded DNA breaks by interfering with homologous recombination repair

**DOI:** 10.18632/oncotarget.3728

**Published:** 2015-03-30

**Authors:** Upadhyayula Sai Srinivas, Jerzy Dyczkowski, Tim Beißbarth, Jochen Gaedcke, Wael Y. Mansour, Kerstin Borgmann, Matthias Dobbelstein

**Affiliations:** ^1^ Institute of Molecular Oncology, University Medical Center Göttingen, Germany; ^2^ Department of Medical Stastics, University Medical Center Göttingen, Germany; ^3^ Department of General, Visceral and Pediatric Surgery, University Medical Center Göttingen, Germany; ^4^ Laboratory of Radiobiology & Experimental Radiooncology, University Medical Center Hamburg-Eppendorf, Hamburg, Germany; ^5^ Tumor Biology Department, National Cancer Institute, Cairo University, Cairo, Egypt

**Keywords:** colorectal cancer, radiochemotherapy, homologous recombination repair, 5-fluorouracil, Rad51

## Abstract

Malignant tumors of the rectum are treated by neoadjuvant radiochemotherapy. This involves a combination of 5-fluorouracil (5-FU) and double stranded DNA-break (DSB)-inducing radiotherapy. Here we explored how 5-FU cooperates with DSB-induction to achieve sustainable DNA damage in colorectal cancer (CRC) cells. After DSB induction by neocarzinostatin, phosphorylated histone 2AX (γ-H2AX) rapidly accumulated but then largely vanished within a few hours. In contrast, when CRC cells were pre-treated with 5-FU, gammaH2AX remained for at least 24 hours. GFP-reporter assays revealed that 5-FU decreases the efficiency of homologous recombination (HR) repair. However, 5-FU did not prevent the initial steps of HR repair, such as the accumulation of RPA and Rad51 at nuclear foci. Thus, we propose that 5-FU interferes with the continuation of HR repair, e. g. the synthesis of new DNA strands. Two key mediators of HR, Rad51 and BRCA2, were found upregulated in CRC biopsies as compared to normal mucosa. Inhibition of HR by targeting Rad51 enhanced DNA damage upon DSB-inducing treatment, outlining an alternative way of enhancing therapeutic efficacy. Taken together, our results strongly suggest that interfering with HR represents a key mechanism to enhance the efficacy when treating CRC with DNA-damaging therapy.

## INTRODUCTION

Colorectal cancer (CRC) is the third most prevalent cancer type in the world [1] and fourth leading cause of cancer related deaths [2]. The cornerstone for the current treatment of rectal cancer, besides surgery, consists in radiochemotherapy i.e. combining anti-cancer drugs and ionizing radiation before and after the surgical removal of the tumor. Radiochemotherapy of colorectal cancers generally includes 5-fluorouracil (5-FU) in combination with γ-radiation.

5-FU is a base analogue that has been in use as an anti-cancer drug for nearly five decades [3]. Apart from CRC, it is used for treating head and neck cancer and breast cancers and others [3]. 5-FU is metabolized upon cellular uptake resulting in (a) misincorporation of FdUTP into DNA, (b) misincoporation of FUTP into RNA and, (c) FdUMP mediated inhibition of thymidylate synthetase (TS).

Endogenous (e. g. reactive oxygen species) and exogenous (e. g. radiation or chemical mutagens) factors can cause DNA damage, and timely repair of the damaged DNA is necessary to maintain genomic stability. Errors during DNA replication lead to replication fork stalling and replicative stress, a condition particularly prevalent in tumor cells [4, 5]. Overexpression or activation of genes that enhance proliferation, such as cyclin E and Ras [6, 7], or treatment with nucleoside analogs, hydroxyurea, or 5-FU [8-10] further exacerbate this stress condition [11]. Replicative stress represents a challenge to the stability of the genome; therefore, the identification and resolution of the damage in a replicating DNA are mandatory for cell survival. A cell accomplishes this by a complex and intricate signaling system known as the DNA damage response (DDR).

The DDR is a coordinated signaling cascade that is activated upon DNA damage and invokes a variety of proteins that help the cell to either repair the damaged DNA or to undergo apoptosis. Two key protein kinases, ATM and ATR, modulate the activity of a vast variety of proteins involved in the DDR. ATM primarily deals with double stranded DNA breaks, while ATR has been associated with single stranded DNA breaks and replicative stress signaling [12]. Around 700 substrates are believed to be phosphorylated by these two master regulatory kinases [13]. One of the substrates of ATM is H2AX, a histone H2A variant that constitutes around 10% of the total H2A in a cell [14]. On formation of DNA double strand breaks (DSB), it is phosphorylated at Ser139, and this phosphorylated H2AX is referred to as γ-H2AX. The phosphorylation of H2AX quickly spreads on the chromatin and can cover mega bases of DNA adjacent to the site of DNA damage [14].

Recently, studies have been conducted to elucidate the 5-FU-induced damage signaling in colorectal cancer derived cell lines. Treatment of HT-29 cells with 5-FU causes activation of Chk1 and Chk2 by ATR and ATM, respectively. HCT 116 cells, irrespective of their TP53 status, showed activation of ATM and Chk2; in HCT15 cells ATM was activated on treatment with high doses of 5-FU [15]. While these studies identified some of the 5-FU-induced damage signaling components, they do not clarify whether and how 5-FU is capable of sensitizing CRC cells towards double strand breaks, as resulting from ionizing radiation. However, such knowledge would be required to understand how 5-FU and irradiation cooperate in the clinical situation of neoadjuvant rectal cancer therapy.

The DDR often triggers repair mechanisms to remedy DNA damage. DNA double strand breaks are recognized by the MRN complex, which promotes the activation of ATM and the phosphorylation of H2AX and other signaling intermediates. Phosphorylated H2AX recruits proteins involved in DNA repair [16]. The DNA double strand breaks can be repaired either by homologous recombination repair (HRR) or the non-homologous end joining (NHEJ). The choice of the repair pathway depends the phase of the cell cycle in which the repair process is occurring and on regulatory factors like 53BP1 and BRCA1 [17, 18]. The fact that many cancers overexpress components of the HRR machinery [19-23] raises the possibility of targeting such components for cancer therapy.

In this study, we show that 5-FU in combination with neocarzinostatin (NCS) causes more sustained DNA damage than the single drugs. The combination also impaired the proliferation of CRC cells more severely than either drug alone. Furthermore, 5-FU reduced the repair of double strand DNA breaks by homologous recombination repair. The Rad51 inhibitor, B02 enhanced the efficacy of radiomimetic treatment similar to 5-FU, suggesting Rad51 inhibitors as alternative radiosensitizers.

## RESULTS

### γ-H2AX persists in cells treated with 5-FU and neocarzinostain

**Figure 1:**
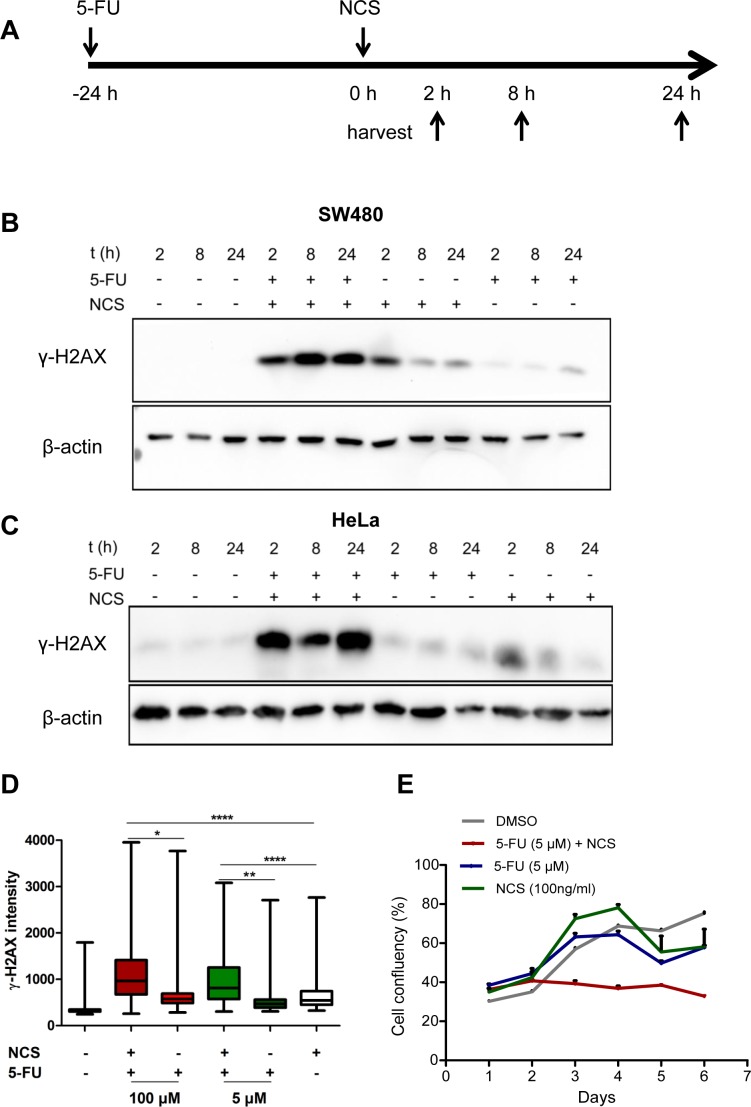
5-FU in combination with NCS leads to persistent γ-H2AX accumulation and decreased survival (A) Schematic representation of how the cells were treated. The cells were first incubated with 5 μM 5-FU for 24 h followed by treatment with 100ng/ml NCS. The cells were then harvested at the indicated time points. (B) SW480 and (C) HeLa cells were treated as described with 5-FU and NCS. Whole cell extracts were immunoblotted, followed by detection of phosphorylated H2AX (γ-H2AX) and β-actin (loading control). (D) SW480 cells were treated with the indicated 5-FU concentrations for 24 h followed by NCS for 24 h. γ-H2AX intensities were determined in single cells by immunofluorescence and automated microscopy. The fluorescence intensities of at least 1000 cells per sample were determined by digital image analysis and are shown in boxplots to indicate the 10^th^, 25^th^, 50^th^, 75^th^ and 90^th^ percentiles (n=3). (E) SW480 cells were treated as described in (D). Cell confluency was measured at regular intervals of 24 h using automated microscopy.

To model neo-adjuvant radiochemotherapy in cell culture, we treated colorectal cancer cells of the line SW480 or HeLa cells with a combination of the base analogue 5-FU and the radiomimetic neocarzinostatin (NCS). We pretreated the cells with 5 μM 5-FU for 24 h followed by addition of 100 ng/ml NCS for 2, 8 and 24 h and immunoblot analysis (Figure 1A-1C). We found that the combination of 5-FU and NCS led to prolonged and persistent γ-H2AX accumulation, even 24 h post NCS treatment. For cells treated with NCS alone, we observed a spike in the γ-H2AX levels at the 2 h time point which diminished over time, suggesting the repair of the damaged DNA (Figure 1B and 1C). Analogous results were obtained when using additional colorectal cancer cell lines, i. e. HCT116, SW837 and SW620 (Supplemental Figure S1, A-E). Next, we treated SW480 cells as above for 24 h and then stained γ-H2AX in fixed cells by immunofluorescence, followed by quantification of the staining intensity by automated microscopy and image analysis (Figure 1D). Here again, we found persistent γ-H2AX when combining 5-FU and NCS. A similar cooperation was observed when varying the concentrations of 5-FU (Figure S2A) and NCS (Figure S2, B-D). These observations raised the question how 5-FU prolongs the DNA damage induced by NCS; we suspected that 5-FU might interfere with the repair of double stranded DNA breaks and thereby increase the toxicity of DSBs. In order to evaluate the effect of 5-FU and NCS treatment on long term cell proliferation and survival, we monitored cell clonogenicity using bright field microscopy along with automated image analysis. As shown in Figure 1E, combined treatment with 5-FU and NCS severely reduced overall cell proliferation. On the other hand, single treatments with 5-FU and NCS allowed cells to recover upon removal of the drug. This indicated that the damage induced by the combination of 5-FU and NCS is severe and more sustainable than upon single drug treatment.

### Homologous recombination repair is reduced upon 5-FU treatment

**Figure 2:**
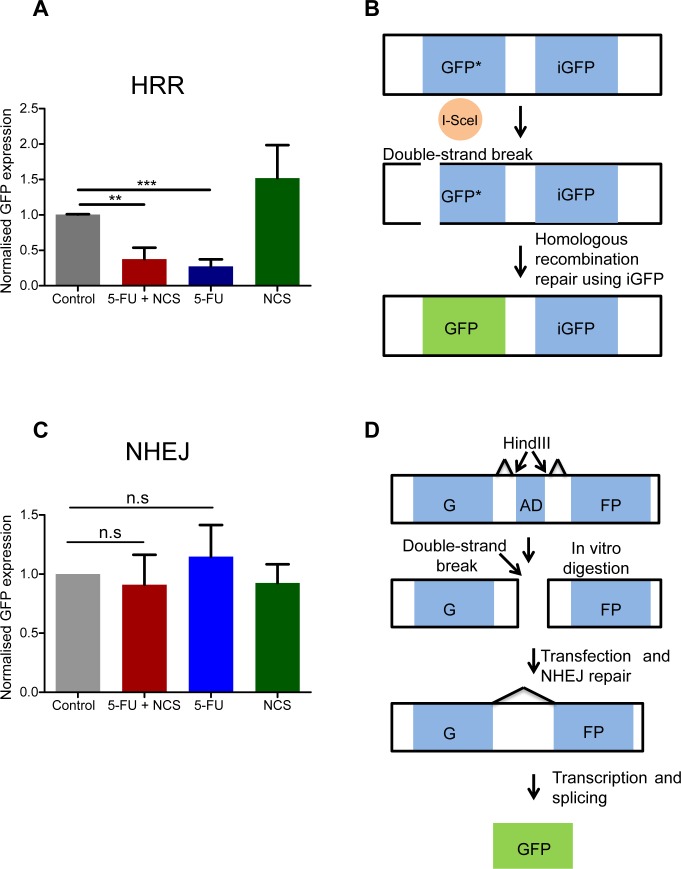
5-FU reduces the homologous recombination repair (A) DRGFP assay to assess the HRR. The normalized GFP intensity, as determined by flow cytometry upon transfection of reporter plasmids, to assess the activity of HRR upon drug treatment. GFP positive cells were measured using flow cytometer and normalized to the control. Note that these assays require extreme transfection efficiencies and were therefore performed only in HeLa cells. (B) Schematic representation of the DRGFP assay. The plasmid contains two non-functional, truncated GFP cassettes. The cassette at the 5′ end has an I-SceI restriction endonuclease cleavage site. The synthesis of I-SceI in the cell by the virtue of a closed circular transfected expression plasmid produces a double strand break in the GFP cassette as shown. If the cell uses the GFP cassette at the 3′ end (iGFP) to repair the double strand break using HRR a functional GFP cassette is generated. (C) NHEJ assay in analogy to A. GFP positive cells were measured using a flow cytometer and normalized to the control. Note that these assays require extreme transfection efficiencies and were therefore performed only in HeLa cells (D) Schematic representation of the NHEJ assay. The NHEJ plasmid contains two exons that together encode GFP, separated by an adenovirus derived (AD) exon. The Hind III restriction endonuclease sites are present on either side of the AD exon, as depicted in the figure. The plasmid was first digested with Hind III *in vitro* and then transfected in to HeLa cells. If the cell repairs the damage using NHEJ, the splice donors and acceptor sites are so placed that a functional GFP transcript is produced. Results from at least three independent experiments are shown with columns representing the standard error of the mean.

NCS, like γ-radiation, primarily induces double strand DNA breaks. The persistent accumulation of γ-H2AX suggested that 5-FU might interfere with at least one of the principal repair mechanisms for DSBs, HRR or NHEJ. To assess this directly, we carried out plasmid based reporter assays. These assays are based on the repair of a GFP-encoding DNA upon transfection of reporter plasmids [24, 25]. Strikingly, we found that 5-FU reduces homologous recombination repair (Figure 2A-2B). In contrast, we did not observe any significant change in NHEJ efficacy upon treatment with either 5-FU or NCS (Figure 2C-D). We conclude that 5-FU treatment reduces the efficiency by that a cell carries out HRR, whereas the mechanisms of NHEJ remain largely unaffected.

### 5-FU treatment does not impair recruitment of RPA2 or Rad51

**Figure 3:**
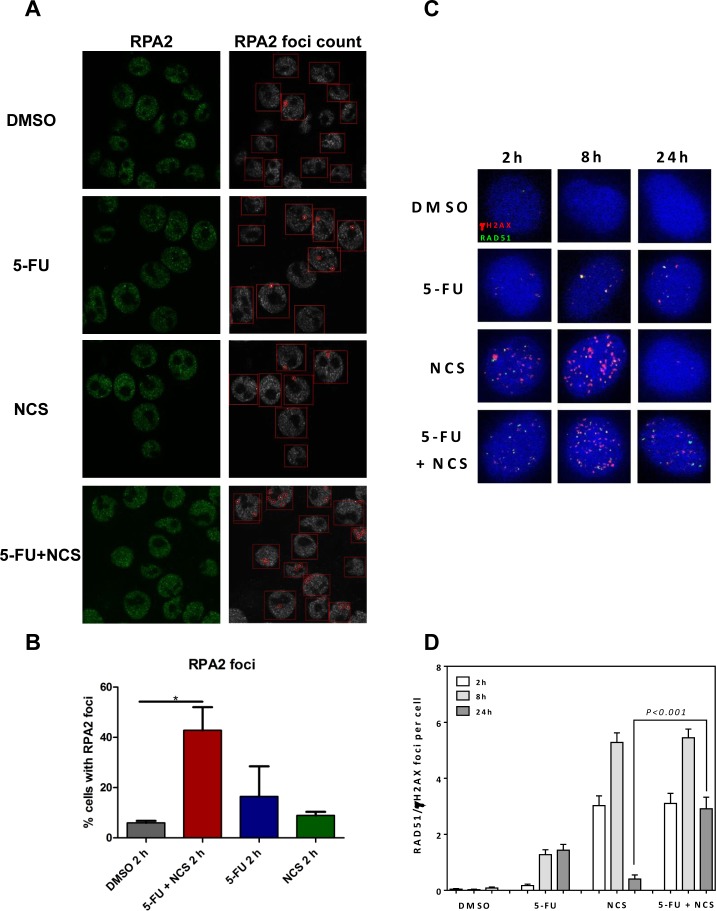
5-FU and/or NCS do not decrease the recruitment of RPA2 and Rad51 (A) Confocal microscopy images of SW480 cells treated with, DMSO, 5 μM 5-FU + 100ng /ml NCS, 5 μM 5-FU, and 100 ng/ml NCS for 2 h (B) RPA2 foci were counted in at least 100 cells per sample per experiment (n=3), and the percentage of cells containing detectable foci were plotted as a histogram. The RPA2 foci were counted using the FociCounter software, (C) Representative IF images for Rad51 colocalized with γH2AX foci at the indicated time points after treatment with DMSO, 5 μM 5-FU + 100ng /ml NCS, 5 μM 5-FU, and 100 ng/ml NCS for 2, 8 and 24 h, (D) Quantification of Rad51 colocalized with γH2AX foci counted in at least 100 cells each at the indicated time points. Error bars represent the SEM of two independent experiments.

In principle, impaired factor recruitment could represent one reason for reduced HRR. One group of such factors is represented by the RPA complex. We therefore assessed the recruitment of RPA2, a subunit of the RPA complex, to see if 5-FU treatment interferes with early stages of HRR. 5-FU did not impair the recruitment of RPA2 to the intranuclear structures, as indicated by the foci formation of RPA2 detected by immunofluorescence and digital image analysis (Figure 3A and 3B). On the contrary, treatment with 5-FU and NCS together led to an increase in RPA2 foci formation 2 h post NCS treatment. The more pronounced foci formation 2 h post NCS treatment is in accordance with the extensive DNA damage induced by 5-FU in combination with NCS. At longer times post NCS treatment, i. e. 8 and 24 h, cells that were pretreated with 5-FU showed even more pronounced RPA2 foci and more discrete colocalization with γ-H2AX (Supplemental Figure S3 A-C). It can therefore be concluded that 5-FU does not impair the recruitment of RPA2. We next investigated the recruitment of Rad51, a key component of HRR, in response to treatment with 5-FU and NCS (Figure 3C and 3D, and Supplemental Figure S4). Treatment with NCS alone led to the formation of Rad51 foci that mostly colocalized with γ-H2AX foci at 2 h post treatment and increased further at 8 h post treatment before being reduced to near-background levels 24 h post treatment. In contrast, treatment with a combination of 5-FU and NCS gave rise to sustained Rad51 foci 24 h post NCS treatment. We conclude that 5-FU does not impair the recruitment of repair proteins, like RPA2 and Rad51. This suggests that 5-FU-induced HRR inhibition does not occur through interference with the early steps of HRR.

### Reduced homologous recombination is not caused by loss of cells in S or G2 phases of the cell cycle

**Figure 4:**
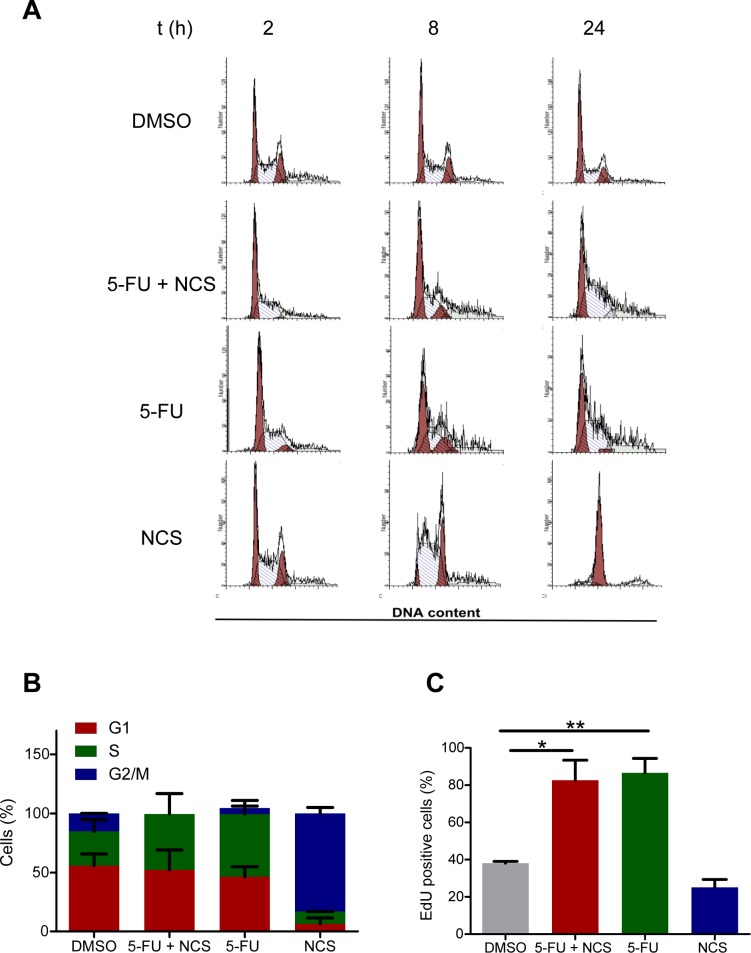
Reduced HRR is not a consequence of a shift in cell cycle distribution (A) Propidium iodide staining and flow cytometry of SW480 cells treated with DMSO, 5 μM 5-FU and/or 100 ng/ml NCS as detailed in Figure 1A. (B) Quantification of the cell cycle distribution. (C) EdU assay in SW480 cells to determine the percentage of cells in S phase. The cells were first treated as shown in Figure 1A; 2 h prior to fixation, 15 μM EdU was added to the cells. The cells were then fixed on the plate, and EdU was detected by Alexa 488 azide. Fluorescence intensity was quantified as described for γ-H2AX in Figure 1C.

Double strand break repair processes are cell cycle dependent. Most of the NHEJ is restricted to the G1 phase, while HRR is active in the S and G2 phases [26]. We therefore assessed whether the reduction in homologous recombination was a consequence of a shift in the cell cycle phase distribution upon treatment with 5-FU. To this end, we performed propidium iodide staining and flow cytometry of SW480 cells (Figure 4A) and found that 5-FU actually increases the proportion of cells in S phase (Figure 4B). This was further confirmed by the detection of DNA synthesizing cells through 5-ethynyl-2′deoxyuridine (EdU) incorporation. Indeed, 5-FU treatment increased the fraction of EdU positive cells (Figure 4C) This indicated that the observed reduction in HRR upon 5-FU treatment did not occur due to arrest of the cells in an unfavorable cell cycle phase. The accumulation of cells in S phase upon 5-FU treatment is compatible with active HRR. Nonetheless, 5-FU leads to a reduction in this repair process, possibly through interference with DNA polymerization during HRR.

### Antagonizing homologous recombination repair by a Rad51 inhibitor also leads to persistence of DNA damage

**Figure 5:**
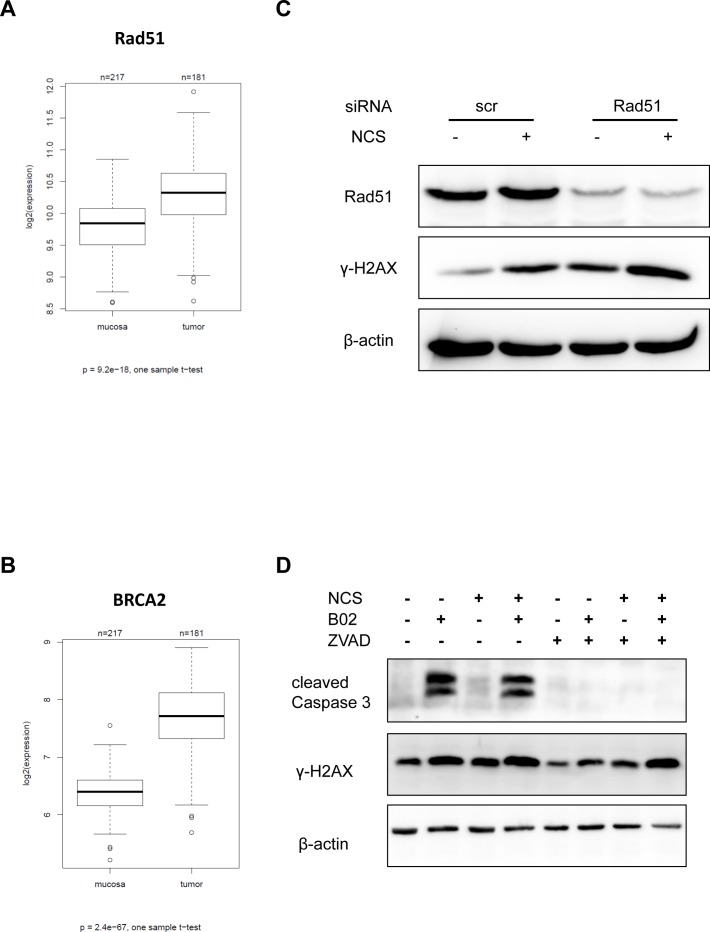
Inhibition or knockdown of Rad51 also leads to persistent γ-H2AX mRNA expression of HRR components in tumors and normal mucosa evaluated in biopsy material from human specimens using microarray hybridization (A) Rad51 and (B) BRCA2. (C) SW480 cells were depleted of Rad51 using siRNA for 16 h and then treated with NCS for 24 h. Whole cell extracts were immunoblotted followed by detection by the indicated antibodies. (D) SW480 cells were treated with B02 and/or ZVAD for 24 h followed by NCS for 24 h. Whole cell extracts were immunoblotted, followed by detection by the indicated antibodies. β-actin was used as a loading control.

Among the proteins involved in homologous recombination repair, Rad51 and BRCA2 represent is a key modulators. As a first step to assess their importance in CRC, we determined the RNA levels corresponding to these genes by microarray hybridization of 181 CRC biopsy samples in direct comparison with 215 normal mucosa biopsies. And indeed, both Rad51 and BRCA2 expression levels were upregulated in the tumors to a highly significant extent (Figure 5A-5B; refer Supplemental Table S1 for raw data and patient characteristics). No significant relation was observed between the tumor grade, and expression of Rad51 or BRCA2 (Supplemental Figure S5). The mRNA expression levels of Rad51 and BRCA2 were highest in S phase of the cell cycle (Supplemental Figure S6). Since many tumors contain a high proportion of dividing cells, S-phase-associated expression may partially explain why Rad51 and BRCA2 levels are high in tumors. In any case, HR mediators are enhanced in CRCs and may thus support CRC progression. This prompted us to test whether direct HR inhibitors may sensitize CRC cells towards DSBs, as 5-FU does. To this end, we firstly depleted Rad51, a principal mediator of HR, and found that this strongly augmented the levels of γ-H2AX 24 h after NCS treatment (Figure 5C). Next, we employed the newly developed Rad51 inhibitor B02 [27]. We treated CRC cells with B02, with or without co-treatment with NCS and analyzed the accumulation of γ-H2AX (Figure 5D). We found that cells treated with B02 and NCS showed a strong accumulation of γ-H2AX; however, cells treated with B02 alone did so too. Since it has been previously reported that apoptosis can cause accumulation of γ-H2AX, we next determined the extent of B02- induced apoptosis by detecting cleaved caspase 3. We found that treatment with B02 alone was sufficient to induce apoptosis (Figure 5). To distinguish this caspase-mediated γ-H2AX accumulation from immediate DDR, we inhibited caspase activity by ZVAD-FMK. On simultaneous treatment of cells with B02 and ZVAD-FMK, apoptosis was reduced, as seen by lack of cleaved caspase 3. We now probed for the accumulation of γ-H2AX under these new conditions and found that treatment with B02 alone did not lead to any detectable γ-H2AX accumulation. Importantly however, treatment with B02 and NCS caused massive γ-H2AX accumulation similar to the combination of 5-FU and NCS together, even when the caspases were blocked (Figure 5D). These results strongly argue that Rad51 inhibition hinders the repair of ds DNA breaks. This raises the perspective of using Rad51 inhibitors to radiosensitize rectal cancer cells, especially in the context of cancer cell resistance towards 5-FU.

## DISCUSSION

Our results strongly suggest that 5-FU sensitizes colorectal cancer cells towards double stranded DNA breaks (DSBs), by interfering with the repair of such lesions. This finding provides a rationale for the widely used radiochemotherapy regimen, including 5-FU and ionizing irradiation, and applied as a neoadjuvant strategy to treat rectal cancer. Mechanistically, 5-FU interferes with homologous recombination repair (HRR) but not non-homologous end joining (NHEJ) (Figure 2). Although a majority of 5-FU-treated cells remains in cell cycle phases where the HRR machinery would be available to the cell (Figure 4), this repair pathway still fails to eliminate DNA damage. Indeed, 5-FU-treated cells, when exposed to a DSB-inducing agent, accumulate RPA2 at the damaged site (Figure 3), but nonetheless, the lesions persist. We therefore propose that 5-FU does not block the assembly of the HRR machinery but instead interferes with the subsequent steps of HRR. Namely, the polymerization of DNA in the course of HRR may be hampered by the misincorporation of 5-FU metabolites (5F-dUTP), or by perturbing the balance of nucleotide pools through the inhibition of thymidylate synthetase [3].

Interestingly, we observed that, although 5-FU interferes with HRR (Figure 2A), it affects long term cell proliferation only mildly (Figure 1E), while a combination of 5-FU and NCS severely impairs it (Figure 1E). These results can be viewed as evidence that 5-FU-induced DNA damage is low and does not challenge the cellular repair machinery with irreparable DNA damage. On the other hand, the 5-FU-induced reduction in HRR makes it impossible for the cell to meet the need of repair when extensive double strand DNA breaks are induced by NCS; this then results in prolonged DNA damage (Figure 1B-1D), and impaired cell proliferation (Figure 1E). This also suggests that the transient suppression of HRR by 5-FU is as such well tolerated by tumor cells, but sensitizes them towards the additional induction of double strand DNA breaks. Our results also give credence to the presently used clinical regimen of combining 5-FU based chemotherapy with radiation.

These results not only provide an explanation why combining 5-FU with ionizing irradiation in cancer treatment. On top of this, the findings reported here suggest an alternative approach to radiosensitization, especially when cancer cells are found resistant against 5-FU. Resistance gained to 5-FU can be attributed to multiple actors like overexpression of thymidylate synthetase (TS) for overcoming the inhibition by 5-FU, Bcl-2 and Bcl-XL [28] protecting cells from 5-FU-induced apoptosis, or lack of mismatch repair (MMR) factors [29]. In such cases, and according to our results, blocking Rad51 by specific inhibitors appears as a valid alternative to achieve radiosensitization, at least in case of a successful clinical development of Rad51 inhibitors. In addition, however, Chk1/Chk2 inhibitors may also turn out suitable to interfere with HRR and hence for radiosensitization, as has been reported previously. AZD7762, a Chk1 inhibitor prevents the formation of Rad51 foci in pancreatic carcinoma cells [30] and also leads to persistent γ-H2AX in response to γ-radiation. Rad51 is phosphorylated at Thr 309 by Chk1 [31], facilitating its recruitment to the sites of DSBs which may explain why Chk1 inhibition reduces HRR efficiency. Interfering with DNA repair mechanisms appears to represent a broadly applicable route to improve cancer therapy, as has been recently reviewed [32]. Along this line, 5-FU has previously been found to interfere with additional repair pathways as well, in particular base excision repair and mismatch repair [33]. Thus, it is conceivable that 5-FU exerts at least a proportion of its cytotoxic effects, alone or in combination with additional therapeutics, by interfering with multiple DNA repair pathways.

If interfering with HRR radiosensitizes tumor cells, this raises the question whether the levels and activity of HRR mediators might serve as predictive markers for radio- and chemosensitivity. And indeed, our results show that colorectal cancers express higher levels of Rad51 and BRCA2 than normal mucosa (Figure 5). This upregulation can be understood as a compensatory mechanism, selected for due to generally enhanced spontaneous DNA damage in most cancer cells [34]. However, it remains to be determined whether these expression levels in tumor cells also correlate with the response to radiochemotherapy. Rad51 overexpression showed inverse correlation with survival in patients with colorectal cancer and head and neck cancer perhaps arguing against a direct role in tumor cell resistance to therapy [35, 36]. However, higher levels of Rad51 were found to be associated with enhanced tumor grading of invasive breast ductal breast cancer [20]. Recently, overexpression of Rad51 was found as a negative predictive marker for neoadjuvant therapy using cisplatin/5-FU to treat esophageal cancers again arguing that high Rad51 levels may confer tumor cell resistance towards DNA damaging drugs [37].

Taken together, our results point out that we are already interfering with DNA repair mechanisms for cancer therapy when using conventional chemotherapeutics. Apparently, DNA repair is blocked by 5-FU, and this contributes to the success of radiotherapy. By adding to our understanding on how DNA repair modulates radio- and chemosensitivity, the development of more targeted repair inhibitors may enable greater efficacy and specificity of sensitization. One example for such a strategy appears to consist in the direct inhibition of HRR to improve the neoadjuvant therapy of rectal cancer.

## EXPERIMENTAL PROCEDURES

### Cell culture, transfection, chemicals and treatments

HeLa and SW480 cells were cultured in Dulbecco's modified Eagle's medium (DMEM) and RPMI 1640, respectively (Invitrogen) supplemented with 10% fetal calf serum and antibiotics. For siRNA-mediated knockdown, cells were reverse transfected with 10 nM pre-designed Silencer Select siRNAs (Invitrogen) using Lipofectamine 2000 (Invitrogen). Cells were treated with the chemotherapeutics, 5-Fluorouracil (5-FU, F6627, SigmaAldrich), Neocarzinostatin (NCS, N9162, Sigma -Aldrich), B02 (SML 0634, SigmaAldrich), and ZVAD-FMK (V116, SigmaAldrich). The samples treated with the Dimethyl sulfoxide (DMSO, A3672, Applichem) served as negative controls.

### Immunoblotting and antibodies

Cell lysates were separated by SDS polyacrylamide gel electrophoresis and transferred to nitrocellulose membranes. For detection of specific proteins, the membranes were incubated with antibodies diluted in tris-buffered saline (TBS) containing 0.1% Tween-20 and 5% bovine serum albumin (BSA). The following primary antibodies were used: mouse anti-γ-H2AX (JBW301, Millipore, Merck), mouse anti-β-actin (AC-15, Abcam), mouse anti-HSC70 (B-6, Santa Cruz Biotechnology), rabbit anti cleaved Caspase 3 (9664, Cell signaling), rabbit anti-Rad51 (H92, Santa Cruz Biotechnology). Primary antibodies were detected with peroxidase-coupled secondary antibodies (Jackson, ImmunoResearch Europe).

### Proliferation assay

For cell proliferation analysis, cells were grown in 96-well plates and treated with 5 μM 5-FU for 24 h, followed by addition of 100 ng/ml NCS and further incubation for 24 h. Then the drugs were removed and cell confluency was monitored every 24 h the using Celigo, an automated microscopy device (Cyntellect). The media was changed every 48 h.

### Flow cytometry

For flow cytometry studies of cell cycle progression, cells were harvested, fixed in 70% ethanol and stained with propidium iodide (PI, P4864, Sigma-Aldrich). Samples were then analysed by a Guava EasyCyte Plus system (Millipore, Merck) and the ModFit LT^TM^ software (Verity Software House).

### DRGFP and NHEJ assay

HeLa cells were cultured in 6 well plates to a confluency of 80% and transfected with the plasmids DRGFP or NHEJ (1.1μg) pCBASce1 for HRR (1.1μg) each combined with DsRed (100 ng) by lipofectamine 2000 for normalization. 4 h after transfection, fresh media was added and cells were treated with 5 μM 5-FU/DMSO for 24 h. This was followed by treatment with 100 ng/ml NCS for 24 h while maintaining the treatment with 5-FU. Following treatment, the cells were harvested, suspended in PBS, and GFP as well as RFP expression was measured by flow cytometry using the Guava EasyCyte Plus system (Millipore, Merck).

### Immunofluorescence microscopy and quantitative assesment

Cells were grown in 96 well plates (Becton Dickinson) for 24 h and then treated with 5 μM 5-FU for 24 h followed by addition of 100 ng/ml NCS and another 24 h incubation. Cells were fixed with 4% paraformaldehyde in PBS, permeabilised with 0.5 % Triton-X-100 and stained with the anti γ-H2AX antibody from Millipore (JBW301, Millipore, Merck). The primary antibody was detected using anti-mouse AlexaFluor-546 (Invitrogen, Life Technologies). Counterstaining of the nuclei was done using Hoechst 33342 (5 μg/mL, Invitrogen, Life Technologies). Images were captured with a Pathway HT Cell Imaging System using the AttoVision image acquisition software (Becton Dickinson) and the fluorescence intensity was quantified per nucleus using the BD Image Data Explorer^TM^ software.

### Rad51 foci staining

Cells grown on cover slips were washed once with cold PBS and fixed with 4% para-formaldehyde/PBS for 10 min. Fixed cells were permeabilised with 0.2% Triton X-100/PBS on ice for 5 min. The cells were incubated overnight with primary antibodies: mouse monoclonal anti-phospho-S139-H2AX antibody (Millipore) at a dilution of 1:300, mouse monoclonal anti-RAD51 antibody (Abcam 14B4) at a dilution of 1:1000. After being washed three times with cold PBS, the cells were incubated for 1 h with secondary anti-mouse Alexa-fluor594 (Invitrogen) at a dilution of 1:500 or anti-rabbit Alexa-fluor488 (Invitrogen) at a dilution of 1:600. The nuclei were counterstained with 4′-6-diamidino-2-phenylindole (DAPI, 10ng/ml). Slides were mounted in Vectashield mounting medium (Vector Laboratories). Immunofluorescence was observed with the Zeiss AxioObserver.Z1 microscope (objectives: ECPlnN 40x/0.75 DICII, resolution 0.44 μm; Pln Apo 63x/1.4Oil DICII, resolution 0.24 μm; EC PlnN 100x/1.3 Oil DICII, resolution0.26 μm and filters: Zeiss 43, Zeiss 38, Zeiss 49). Semi-confocal images were obtained using the Zeiss Apotome, Zeiss AxioCamMRm and Zeiss AxioVision Software.

### Confocal microscopy (RPA2 staining)

Cells were grown on cover slips in 6 well plates (Becton Dickinson) for 24 h and then treated with 5 μM 5-FU for 24 h followed by treatment with 100 ng/ml NCS. Cells were fixed with 4% paraformaldehyde in PBS, permeabilised with 0.5 % Triton-X-100 and stained with the anti RPA2 antibody from Calbiochem (NA18, Calbiochem). The primary antibody was detected using anti-mouse IgG antibody coupled to AlexaFluor-488 (Invitrogen, Life Technologies). Counterstaining of the nuclei was done using Hoechst 33342 (5 μg/mL, Invitrogen, Life Technologies). Images were captured with a Carl Zeiss LSM 510 meta confocal microscope (Germany). The RPA2 foci in the nuclei were counted using the FociCounter software (developed by Anna Jucha, University of Wrocław, Poland) http://focicounter.sourceforge.net/.

### Expression analysis of Rad51 and BRCA2 repair genes

To validate the clinical relevance of our genes, we used an in house generated data set (J. Gaedcke). It is based on 181 rectal tumor biopsies and 181 biopies of matched normal mucosa tissues analysed on Whole Human Genome Microarray 4x44K v2 microarrays. Data were log2 transformed and normalized using quantile normalization. The genes relevant for this study were extracted from the complete microarray data by gene symbol. In case of multiple probes, expression levels of probes of the same gene were found to be highly correlated, and the highest expressed probe was used. Differential expression was compared on the log2 differences of tumour vs. mucosa from the same patients, using a one-sample t-test. The data are provided in Supplementary Table 1.

### Cell synchronization by double thymidine block

For synchronization of cell cultures, thymidine (Sigma-Aldrich) was added to the culture medium at a final concentration of 2 mM for 16 h. Thymidine was washed away by adding pre-warmed culture medium to the cells for 5 min five times. Subsequently, the cells were further incubated for 9 h. A second treatment with 2 mM thymidine for 16 h was performed. After washing away thymidine as before, the cells were released in fresh culture medium.

### RNA extraction and quantitative RT-PCR

For mRNA analysis, RNA was isolated from cells using TRIzol (Invitrogen/Life Technologies). The isolated RNA was reverse transcribed using 25U M-Mul V Reverse Transcriptase and random hexameric and oligo-dT primers. For analysis of cDNA samples, SYBR Green (Invitrogen/Life Technologies) was used for quantitative real-time PCR. The following primers were used: GAPDH, 5′-GAAGGTCGGAGTCAACGGATTTG-3′ and 5′-CAGAGATGATGACCCTTTTGGCTC-3′; Rad51, 5′-CAACCCATTTCACGGTTAGAGC-3′ and 5′-GCTTTGGCTTCACTAATTCCCTT-3′; BRCA2, 5′-GTTGTGAAAAAAACAGGACTTG-3′ and 5′-CAGTCTTTAGTTGGGGTGGA-3′. Data were normalized to GAPDH. Relative gene expression was calculated by using the ΔΔCt method.

### Statistical analysis

All data are represented as means plus/minus standard error. Unpaired Student's t-test was used for the calculation of p-values. Asterisks indicate significant differences (*, p < 0.05; **, p < 0.01; ***, p < 0.001; ****, p<0.0001), n.s. = not significant. *n* in figure legends indicates the number of independent experiments.

## SUPPLEMENTARY MATERIAL, FIGURES, TABLES


