# Supplementary material for: 5-Fluorouracil sensitizes colorectal tumor cells towards double stranded DNA breaks by interfering with homologous recombination repair
Source: Oncotarget. 2015 Mar 30;6(14):12574–86. doi: 10.18632/oncotarget.3728 (PMC4494959; doi:10.18632/oncotarget.3728)
Supplement: Supplementary file 1 [file oncotarget-06-12574-s001.pdf]

# 5-Fluorouracil sensitizes colorectal tumor cells towards double stranded DNA breaks by interfering with homologous recombination repair

**Supplementary Material**

(A)

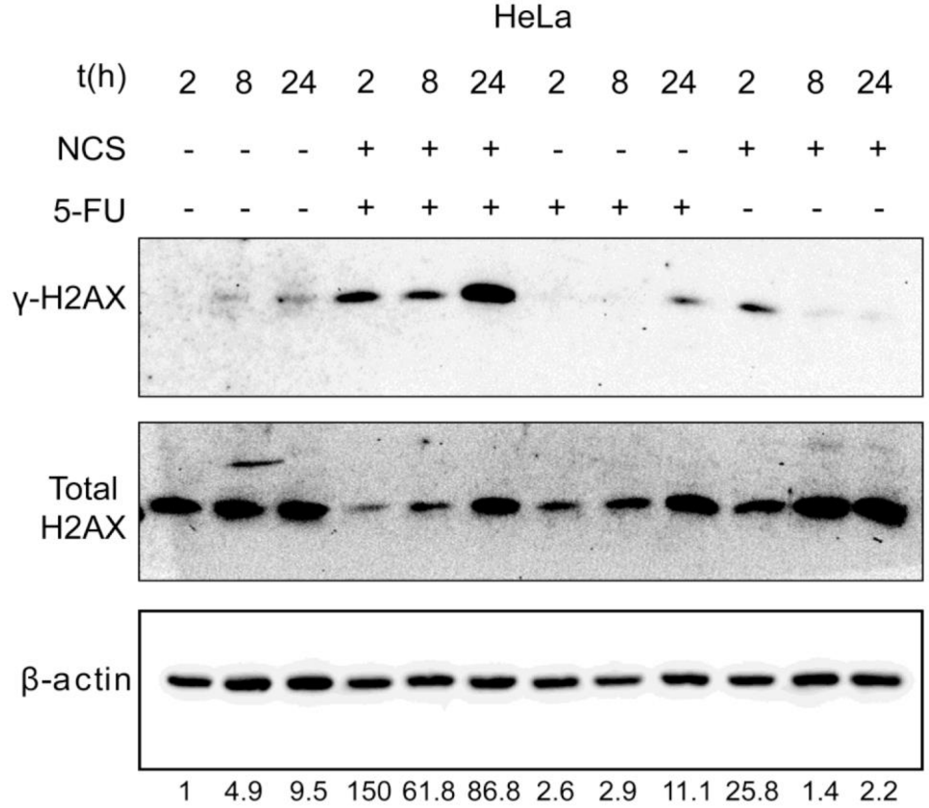
HeLa
t(h) 2 8 24 2 8 24 2 8 24 2 8 24
NCS - - - + + + - - - + + +
5-FU - - - + + + + + + - - -
γ-H2AX
Total H2AX
β-actin
1 4.9 9.5 150 61.8 86.8 2.6 2.9 11.1 25.8 1.4 2.2

(B)

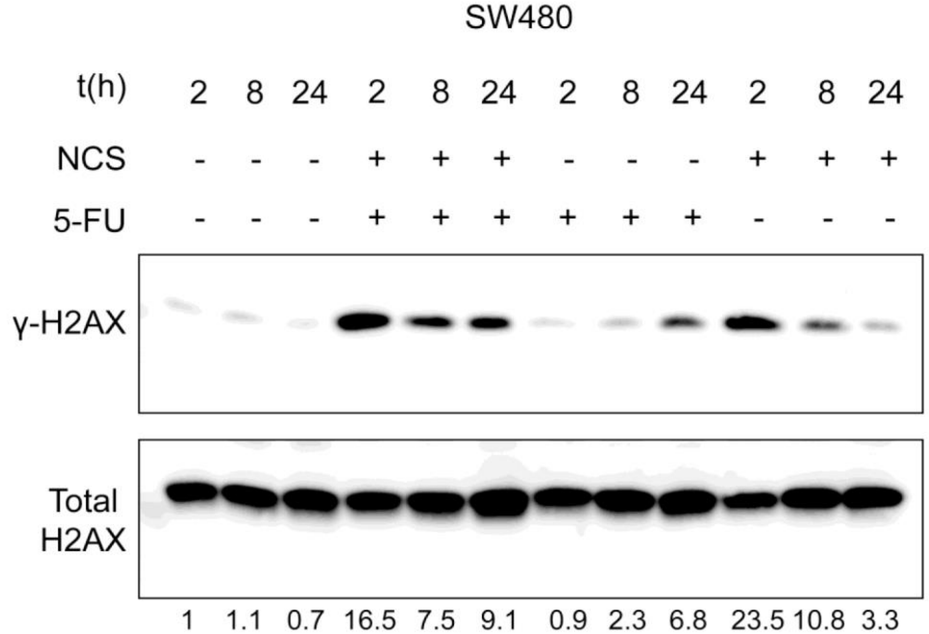
SW480
t(h) 2 8 24 2 8 24 2 8 24 2 8 24
NCS - - - + + + - - - + + +
5-FU - - - + + + + + + - - -
γ-H2AX
Total H2AX
1 1.1 0.7 16.5 7.5 9.1 0.9 2.3 6.8 23.5 10.8 3.3

(C)

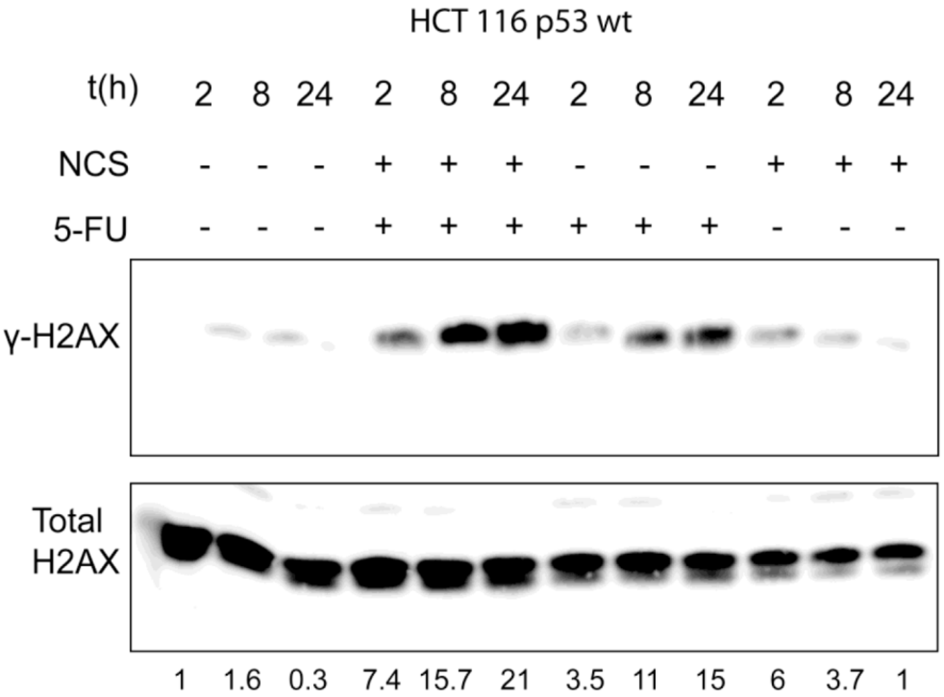
HCT 116 p53 wt
t(h) 2 8 24 2 8 24 2 8 24 2 8 24
NCS - - - + + + - - - + + +
5-FU - - - + + + + + + - - -
γ-H2AX
Total H2AX
1 1.6 0.3 7.4 15.7 21 3.5 11 15 6 3.7 1

(D)

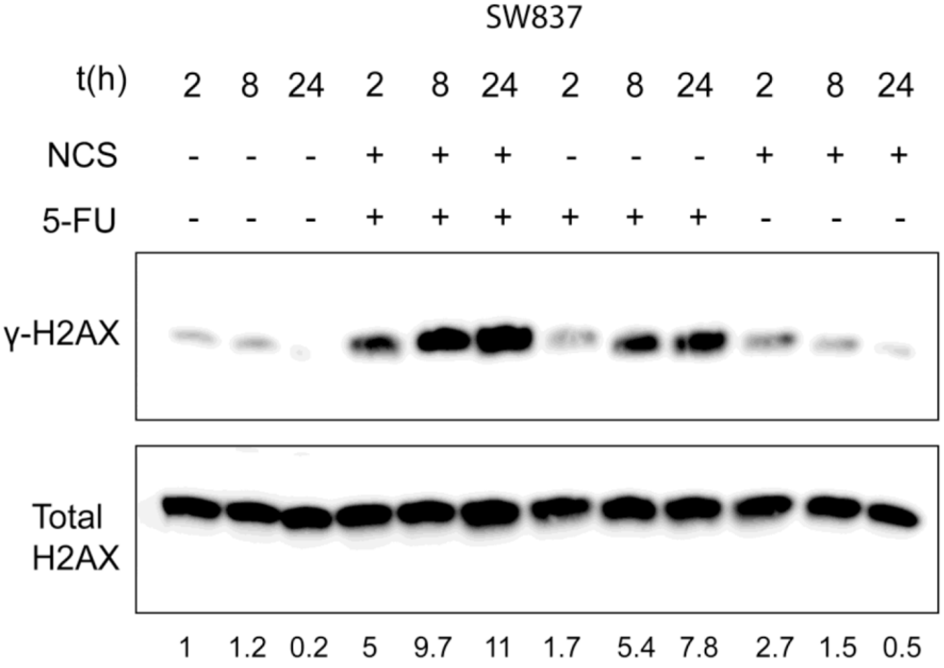
SW837
t(h) 2 8 24 2 8 24 2 8 24 2 8 24
NCS - - - + + + - - - + + +
5-FU - - - + + + + + + - - -
γ-H2AX
Total H2AX
1 1.2 0.2 5 9.7 11 1.7 5.4 7.8 2.7 1.5 0.5

**(E)**

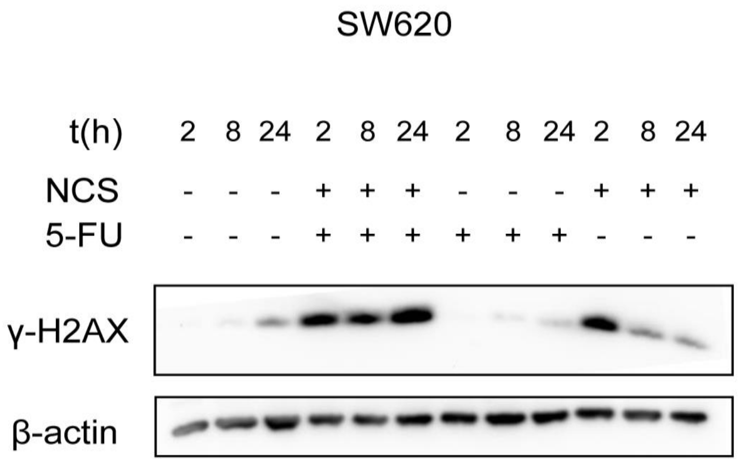


**Supplemental Figure S1**. **5-FU in combination with NCS leads to persistent γ-H2AX accumulation.** Cells were first incubated with 5 µM 5-FU for 24 h followed by treatment with 100ng/ml NCS, and then harvested at the indicated time points. (A) HeLa, (B) SW480, (C) HCT116 p53 wt, (D) SW837 and (E) SW620, cells were treated as described with 5-FU and NCS. Whole cell extracts were immunoblotted, followed by detection of phosphorylated H2AX (γ-H2AX), total H2AX (for normalization of γ-H2AX) and β-actin (loading control).

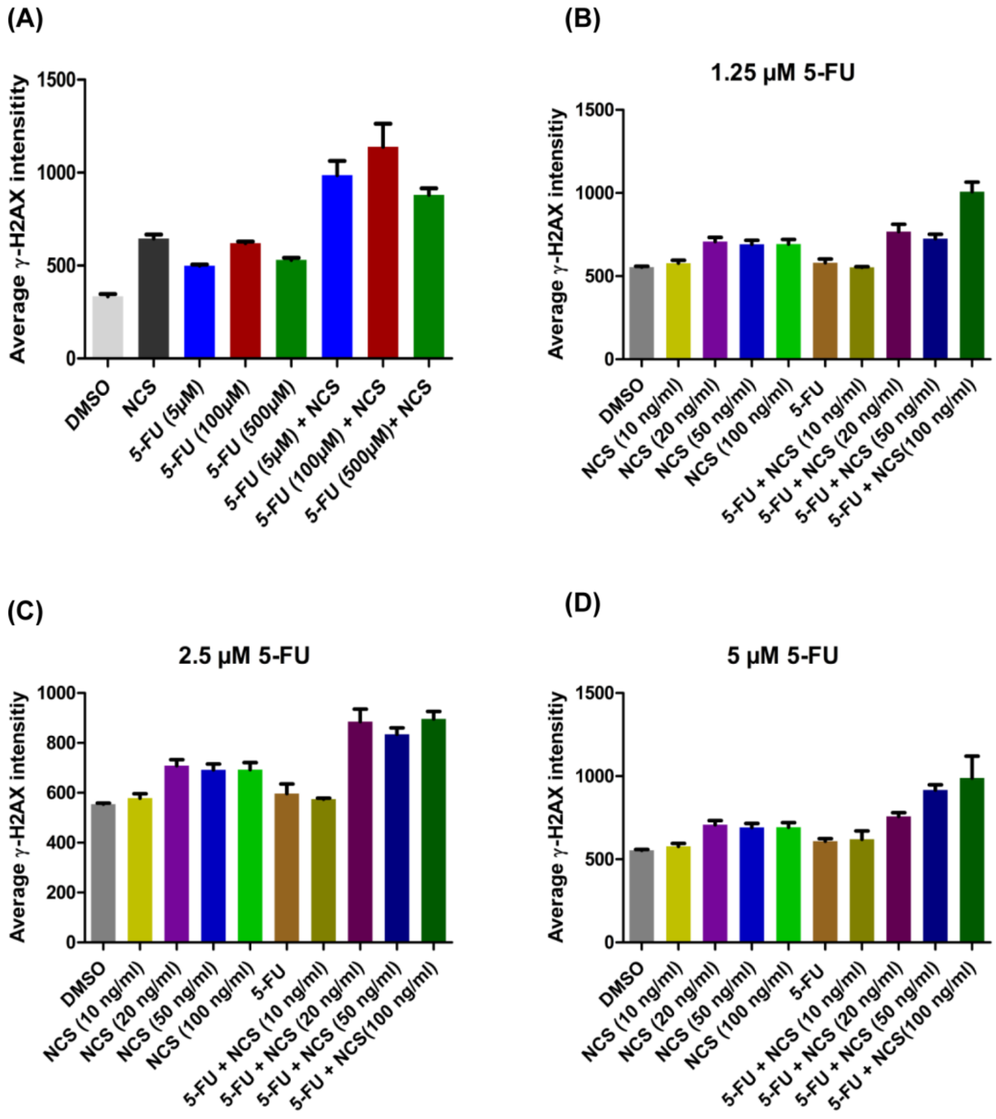


**Supplemental Figure S2. Dose-dependence of DNA damage upon treatment with 5-FU and NCS.** SW480 cells were treated with (A) 100 ng/ml NCS and 5, 100 or 500 µM 5-FU, (B) 1.25 µM 5- FU and 100, 50, 20 or 10 ng/ml NCS, (C) 2.5 µM 5-FU and 100, 50, 20 or 10 ng/ml NCS, (D) 5 µM 5-FU 100, 50, 20 or 10 ng/ml NCS. The cells were treated with the indicated 5-FU concentrations for 24 h, followed by NCS plus 5-FU for 24 h. Cells treated with DMSO were used as negative controls. The γ-H2AX intensities were determined in single cells by immunofluorescence and automated microscopy. The fluorescence intensities of at least 1000 cells per sample were determined by digital image analysis and plotted as the mean and standard error mean of three experiments.

**(A)**

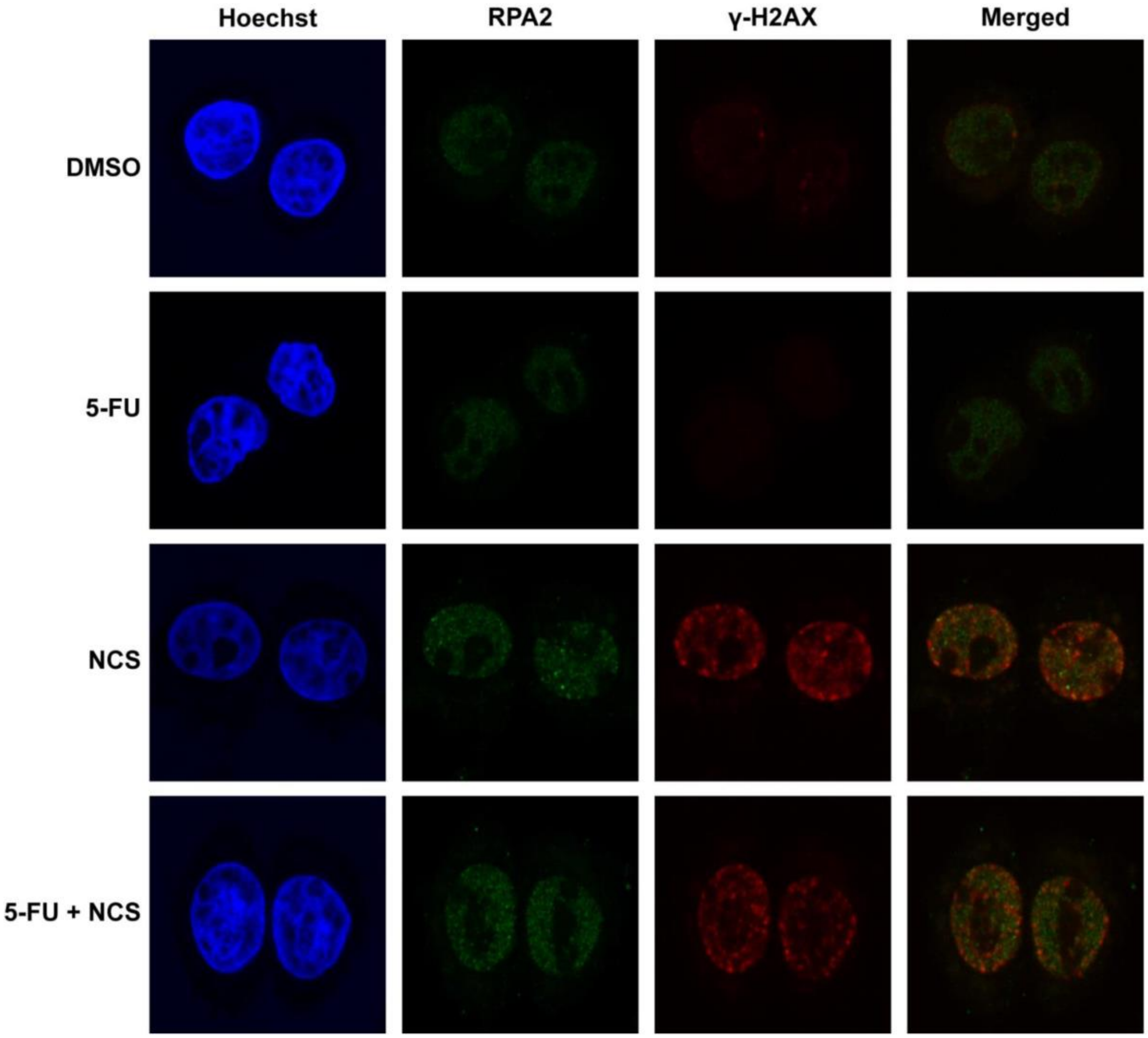

(B)

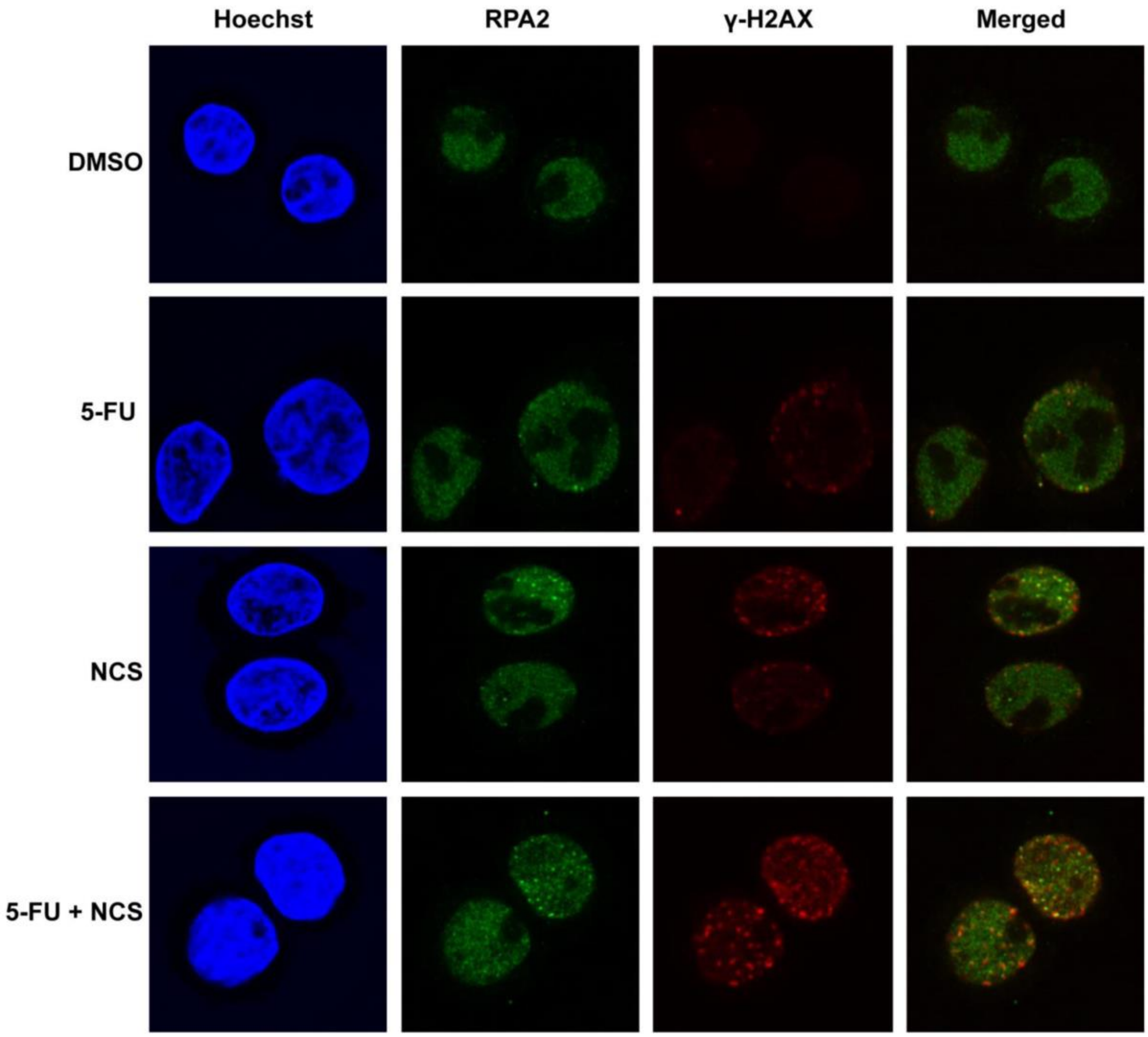

(C)

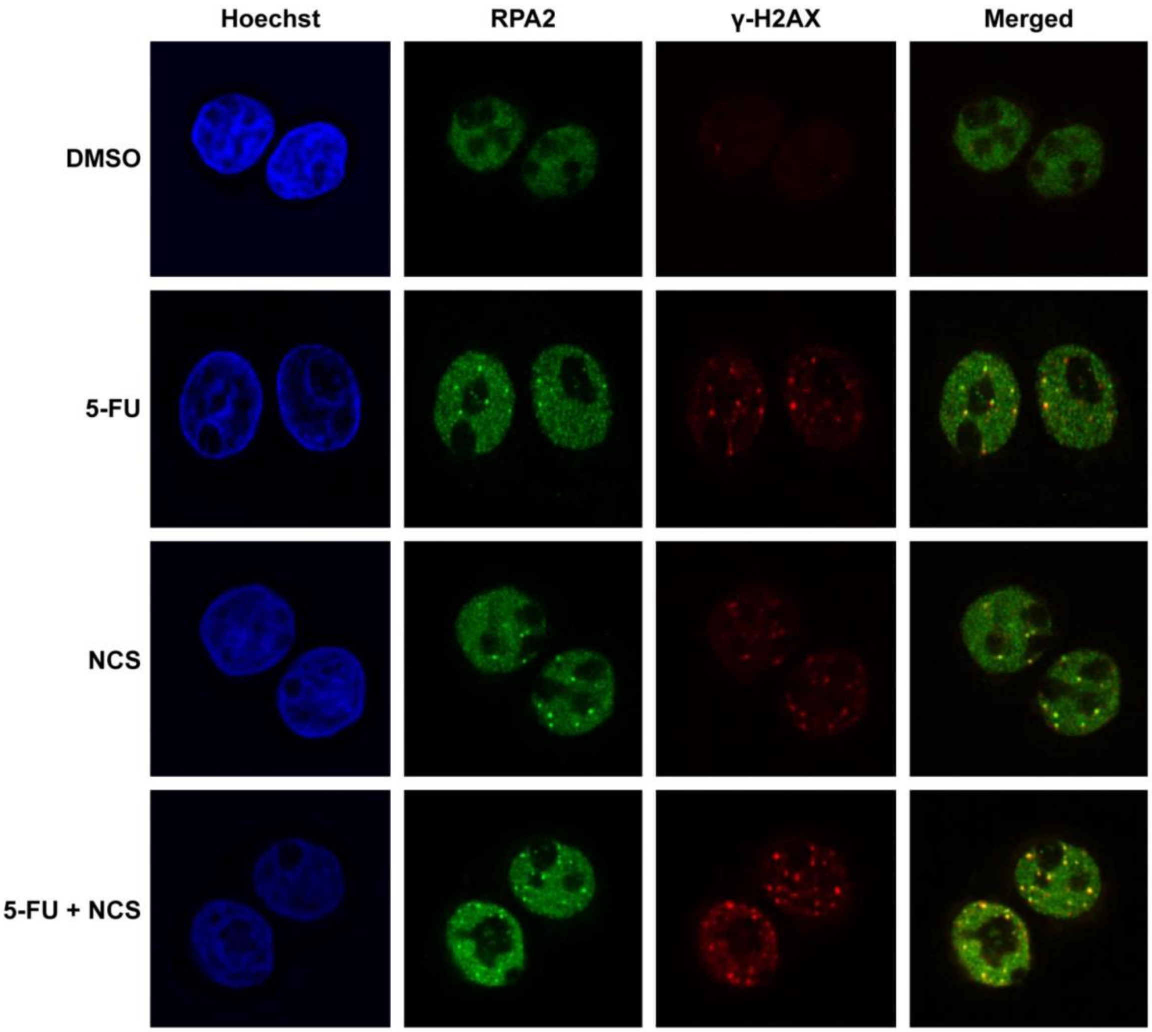


**Supplemental Figure S3. 5-FU and/or NCS do not decrease the recruitment of RPA2.** Confocal microscopy images of SW480 cells treated with DMSO, 5 µM 5-FU + 100ng /ml NCS, 5 µM 5-FU, or 100 ng/ml NCS for (A) 2 h, (B) 8 h and (C) 24 h post NCS treatment, followed by IF staining of RPA2 and phospho-H2AX.

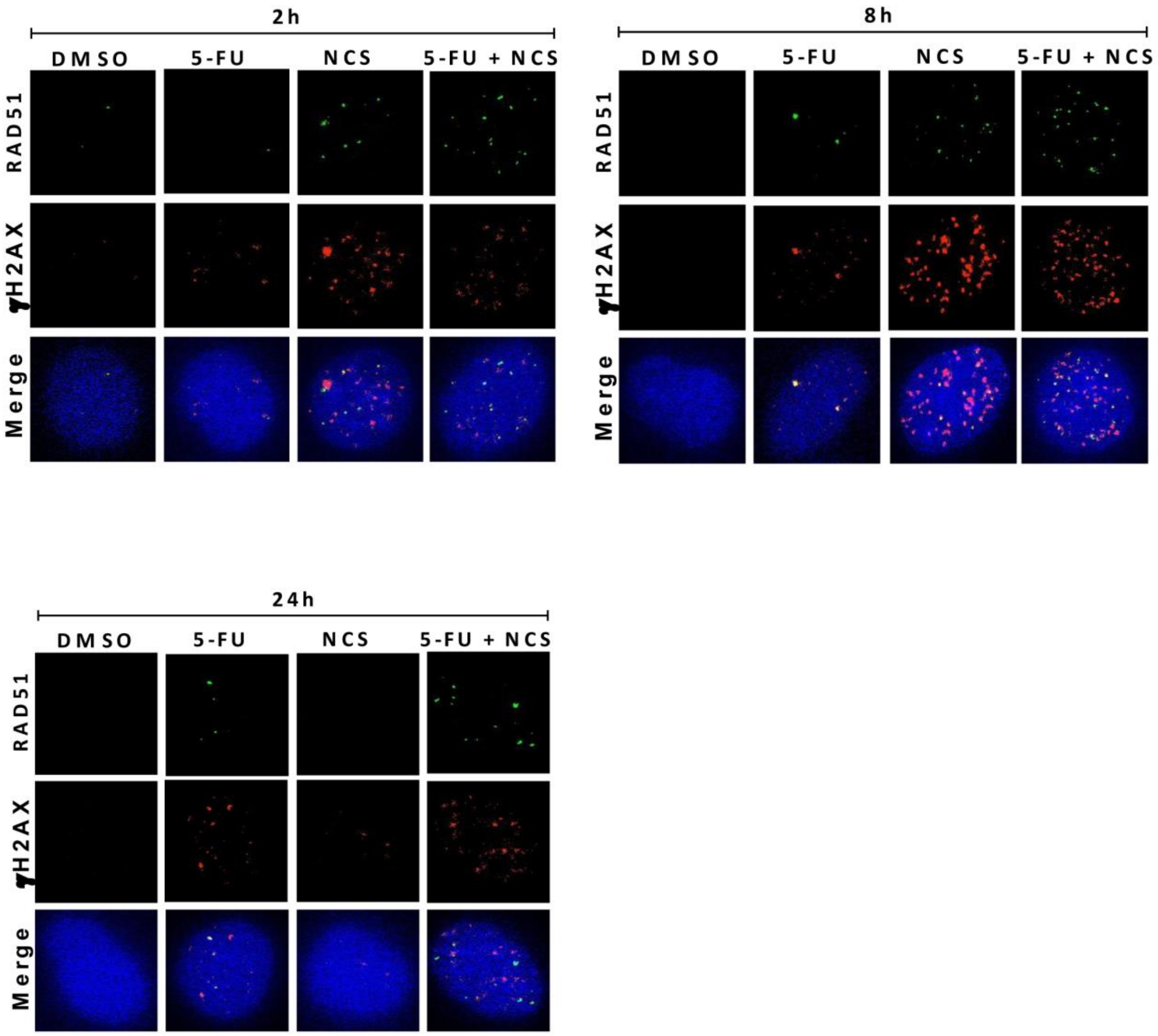


**Supplemental Figure S4. Accumulation of Rad51 and phospho-H2AX foci upon treatment with 5-FU and/or NCS**. SW480 cells were treated and subjected to immunofluorescence analysis as described in Fig. 3, panel C. Separate image files are shown to document the localization of Rad51 and γ-H2AX, and merged images show the partial colocalization of the two.

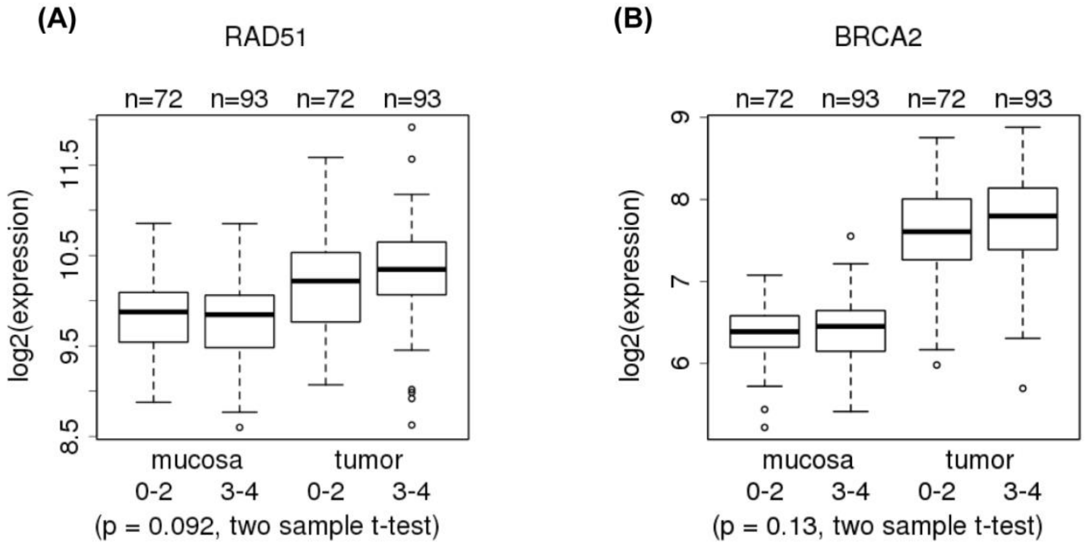


**Supplemental Figure S5**. **Expression of Rad51 and BRCA2 in human biopsies.** mRNA expression of HRR components in colorectal cancers and normal mucosa, evaluated in biopsy material from patients using microarray hybridization, and classified according to tumor grade; (A) Rad51 and (B) BRCA2.

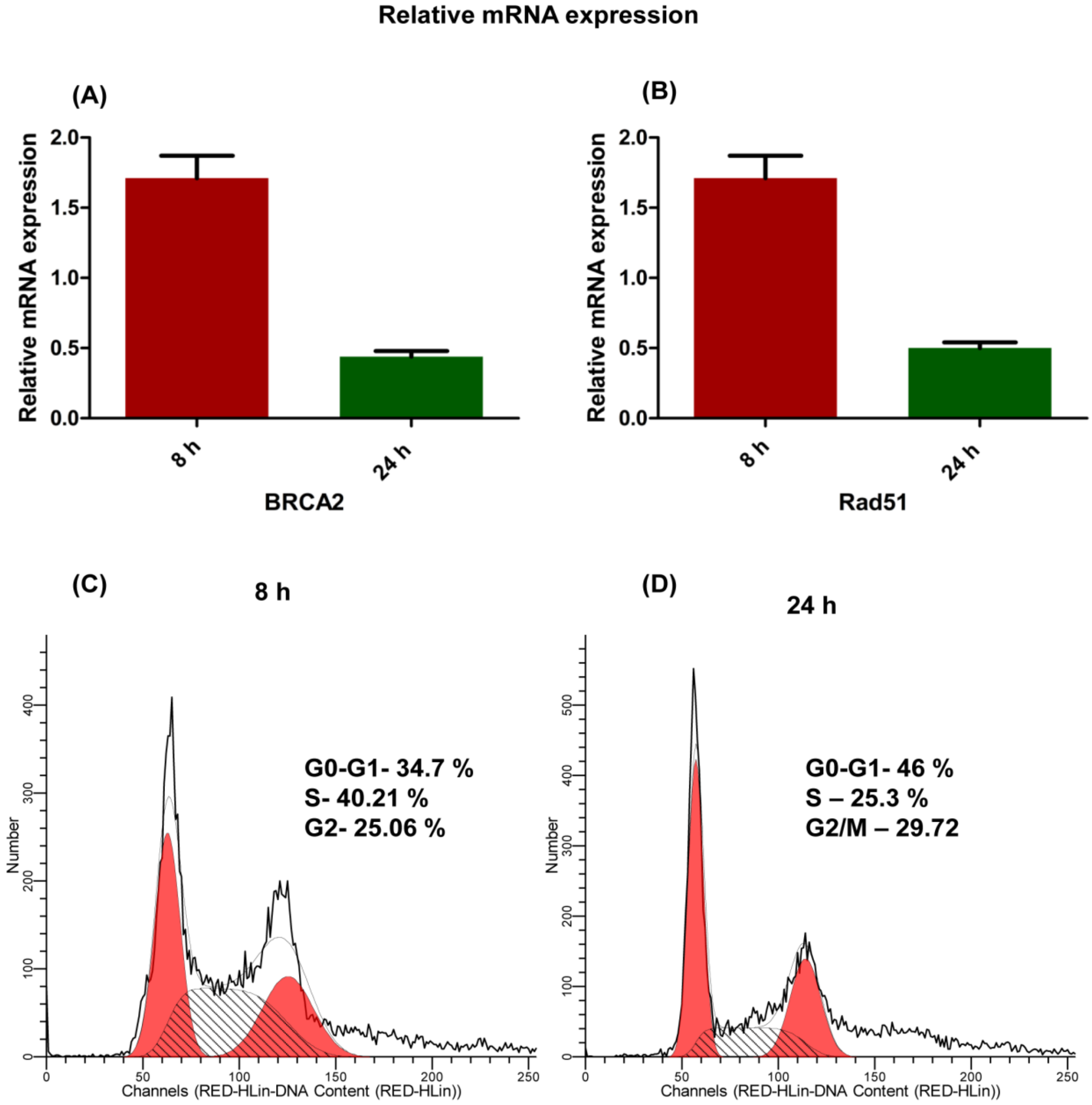


**Supplemental Figure S6. Increased mRNA expression of RAD51 and BRCA2 in S phase** SW480 cells were synchronized using a double thymidine block and then released by removing excessive thymidine. The cells were harvested 8h and 24 h post release, and (A) Rad51 as well as (B)BRCA 2 mRNA levels were analyzed by quantitative RT-PCR and normalized to the reference gene GAPDH. Parallel propidium iodide staining of the cells and analysis by flow cytometry document an enrichment for S phase at 8 h (C) post release and a normal distribution of the DNA content at 24 h post release (D).

| patient | cancer detailed location | gender | age_at_pathologic_diagnosis_by_biopsy_months | age_at_surgery_months | time_to_progression_months | dfs_event | tumor_grade_at_surgery | RAD51 mucosa | RAD51 tumor | BRCA2 mucosa | BRCA2 tumor |
|---|---|---|---|---|---|---|---|---|---|---|---|
| Pat-JG2 | | FEMALE | 663 | 667 | 0.0000000 | TRUE | 3 | 9.93271524271006 | 9.4802183153759 | 6.3207502627855 | 6.88921816365709 |
| Pat-JG4 | | MALE | 783 | 787 | 40.2472330 | FALSE | 4 | 9.98729358974371 | 11.0624710704209 | 6.76901977975552 | 7.30007861171228 |
| Pat-JG6 | | MALE | 747 | 752 | 29.6022506 | FALSE | 4 | 9.30417240819116 | 10.0218374139692 | 6.3133576353198 | 7.8679212132719 |
| Pat-JG8 | | MALE | 737 | 742 | 32.6248999 | FALSE | 4 | 9.3157264466179 | 10.4315478279654 | 6.04772817476927 | 8.17713894890417 |
| Pat-JG10 | | FEMALE | 621 | 625 | 31.5735436 | FALSE | 4 | 9.36872244067524 | 10.2957877158105 | 5.66377074932453 | 7.69047666179671 |
| Pat-JG12 | | MALE | 706 | 709 | 34.4976283 | FALSE | 2 | 9.39162488785565 | 10.5833731474297 | 6.3277349589064 | 8.2808777204461 |
| Pat-JG14 | | MALE | 843 | 847 | 32.6577548 | FALSE | 4 | 10.1179988190345 | 11.1748265004169 | 5.78048749685958 | 7.71506988546085 |
| Pat-JG16 | | MALE | 723 | 727 | 30.2922031 | FALSE | 3 | 9.68299308989286 | 10.7428849346383 | 6.62471156380279 | 8.22605305954173 |
| Pat-JG18 | | MALE | 656 | 660 | 28.0580710 | FALSE | 2 | 9.71652006915316 | 10.5211490270596 | 6.24556446551268 | 7.00267715299219 |
| Pat-JG20 | | MALE | 633 | 637 | 27.5323929 | FALSE | 3b | 9.93773125163652 | 9.96731054193543 | 6.08705501533867 | 7.44525085048825 |
| Pat-JG22 | | MALE | 601 | NA | NA | NA | 4 | 9.35131075560352 | 10.665993515512 | 6.52667352390912 | 8.3969968889138 |
| Pat-JG24 | | MALE | 698 | 702 | 14.9161174 | FALSE | 3 | 9.59710645042617 | 10.9196329992415 | 6.03209814773894 | 6.79329801836578 |
| Pat-JG26 | | MALE | 817 | 821 | 23.3598226 | FALSE | 4 | 9.7293643823097 | 9.70796734910994 | 6.5464411159892 | 7.15618775950004 |
| Pat-JG28 | | MALE | 700 | 705 | 6.7023964 | TRUE | 2 | 9.62580279537653 | 9.84764457558552 | 6.3463263405505 | 8.10491711328965 |
| Pat-JG30 | | MALE | 837 | 841 | 13.0105341 | FALSE | 3 | 10.0156682738079 | 9.8259477888929 | 6.14187050873839 | 6.30552990237904 |
| Pat-JG32 | | MALE | 866 | 869 | 15.6717797 | FALSE | 3 | 9.47165153685243 | 10.5643573523785 | 6.42836936403653 | 8.01147872810892 |
| Pat-JG34 | | FEMALE | 928 | 931 | 34.6947576 | FALSE | 3b | 10.5677266239569 | 10.6748008437323 | 6.77977646409272 | 7.32803477695798 |
| Pat-JG36 | | FEMALE | 819 | 821 | 0.0000000 | TRUE | 2 | 9.73950218360529 | 9.07022979227076 | 5.98788544608275 | 7.46453323914939 |
| Pat-JG38 | | MALE | 899 | 902 | 0.0000000 | TRUE | 3b | 9.69542736716165 | 10.1401927446724 | 6.27058078488092 | 7.23321886432942 |
| Pat-JG40 | | MALE | 912 | 916 | 10.6121276 | FALSE | 1 | 9.99098230674 | 10.228844727222 | 6.18413049755896 | 7.59130113817375 |
| Pat-JG42 | | MALE | 455 | 458 | 8.2465759 | FALSE | 2 | 9.91799057277176 | 10.2069889904269 | 6.27077559834025 | 7.1373413612784 |
| Pat-JG44 | | MALE | 798 | 811 | 31.5078338 | FALSE | | 10.4687577304294 | 10.9553231377159 | 6.6087930095822 | 8.29148266922621 |
| Pat-JG46 | | FEMALE | 732 | 733 | 0.0000000 | TRUE | 1 | 9.03973865887085 | 10.3195374410183 | 5.94255004694169 | 7.32480394784175 |
| Pat-JG48 | | MALE | 829 | 832 | 23.2612579 | FALSE | 1 | 9.53627783683737 | 9.3458615270087 | 5.93924485129285 | 8.754608387631 |
| Pat-JG50 | | MALE | 648 | 650 | 23.7869361 | FALSE | 1 | 9.21040696205692 | 9.70931866209768 | 6.13840681234205 | 6.98701709308822 |
| Pat-JG52 | | MALE | 929 | 932 | 7.7208978 | FALSE | 4 | 10.2236445593094 | 9.96652321665894 | 6.84932353692797 | 7.67308101914763 |
| Pat-JG54 | | FEMALE | 685 | 689 | 7.3266392 | FALSE | 2 | 10.3698435187567 | 9.43098174197723 | 6.90294067079801 | 8.0056090065699 |
| Pat-JG56 | | MALE | 731 | 735 | 15.2446662 | TRUE | 2 | 8.87767253795456 | 9.77605155251985 | 6.05948805740382 | 7.54560738101239 |
| Pat-JG58 | | FEMALE | 674 | 677 | 0.0000000 | FALSE | 2 | 10.0747789549313 | 10.5870451848506 | 6.51474891052344 | 7.50615765688944 |
| Pat-JG60 | | MALE | 561 | 564 | 22.5384505 | FALSE | 2 | 9.72220441411972 | 10.0061693165844 | 6.09618345537365 | 7.83838298054191 |
| Pat-JG62 | | MALE | 749 | 752 | 22.2099016 | FALSE | 4 | 9.17565784725543 | 11.0878743984749 | 6.55933029362852 | 8.03751114666891 |
| Pat-JG64 | | MALE | 611 | 615 | 17.9716216 | FALSE | 4 | 8.76784132791897 | 10.2847470728594 | 6.40495081407863 | 7.38728708524353 |
| Pat-JG66 | | MALE | 876 | 879 | 0.0000000 | TRUE | 1 | 9.57891964331535 | 10.1165797888942 | 6.31671534973123 | 7.04876772525669 |
| Pat-JG68 | | FEMALE | 563 | 566 | 0.0000000 | TRUE | 2 | 9.99695214236266 | 9.66126081285082 | 6.72812776696585 | 7.14485322286778 |
| Pat-JG70 | | FEMALE | 868 | 872 | 16.1974579 | FALSE | 2 | 9.3191225204396 | 10.0612211418024 | 5.86203634025691 | 8.30020317892148 |
| Pat-JG72 | | MALE | 461 | 465 | 6.3738475 | FALSE | 2 | 8.88004739718471 | 10.27171581355 | 5.72174079801253 | 6.96215254950745 |
| Pat-JG74 | | MALE | 902 | 905 | 13.0105341 | FALSE | | 10.0797497360588 | 10.4411512340927 | 6.22342099668726 | 8.66431285381999 |
| Pat-JG76 | | FEMALE | 955 | 960 | 2.9569396 | FALSE | 4 | 9.94515563457634 | 10.5447373712275 | 6.44781798971449 | 8.11600065084322 |
| Pat-JG78 | | MALE | 728 | 731 | 13.0433890 | FALSE | | 9.7895887249862 | 10.901411989869 | 6.15325942922446 | 8.0767564270762 |
| Pat-JG80 | | MALE | 912 | 915 | 1.7413089 | FALSE | 2 | 9.99098230674 | 9.46307981742183 | 6.62782406535132 | 7.49200602207002 |
| Pat-JG82 | | FEMALE | 593 | 593 | 12.5834206 | FALSE | 3 | 9.19913278891553 | 10.1917709560967 | 5.87500438238059 | 8.09292818790451 |
| Pat-JG84 | | MALE | 596 | 599 | 11.5649192 | FALSE | | 9.16991656653822 | 10.8348374266123 | 6.2083808683866 | 8.27596569551153 |
| Pat-JG86 | | MALE | 687 | 691 | 9.8236103 | FALSE | 2 | 9.41037787302977 | 10.512563781673 | 6.02455728660829 | 8.0289920816864 |
| Pat-JG88 | | MALE | 923 | 927 | 8.2794308 | FALSE | 2 | 9.31820896773265 | 10.5328889957611 | 6.26510362756165 | 8.56538962354512 |
| Pat-JG90 | | MALE | 753 | 754 | 0.0000000 | FALSE | | 10.2109309590427 | 10.0855215322497 | 6.14458802391528 | 6.70900099457215 |
| Pat-JG92 | | MALE | 865 | 868 | 22.6698700 | TRUE | 2 | 9.64017203627823 | 10.1296052934051 | 6.04500330733313 | 7.39242862059023 |
| Pat-JG94 | | MALE | 705 | 708 | 0.0000000 | FALSE | 2 | 9.4236289772544 | 9.71905807702446 | 6.34480083310527 | 7.26186810615743 |



| | | | | | | | | | | | |
|---|---|---|---|---|---|---|---|---|---|---|---|
| Pat-JG96 | | MALE | 913 | 915 | 20.1728988 | FALSE | 2 | 9.3748653150296 | 10.5008081443738 | 6.32571072447587 | 7.55746965006727 |
| Pat-JG98 | | MALE | 825 | 828 | 5.8481694 | FALSE | 3 | 9.649334858804 | 10.0966099180031 | 6.50393985618687 | 7.98130671454916 |
| Pat-JG100 | | MALE | 733 | 736 | 0.0000000 | FALSE | | 9.44727731092814 | 10.5373693621352 | 6.27508820379213 | 8.44912566519627 |
| Pat-JG102 | | FEMALE | 793 | 796 | 0.0000000 | FALSE | 1 | 9.61565057818915 | 9.55204383270225 | 6.47756460037906 | 7.74051727603916 |
| Pat-JG104 | | MALE | 783 | 787 | 0.0000000 | FALSE | 4 | 9.5252132425963 | 10.3267670373112 | 6.04014708567165 | 7.12976625832868 |
| Pat-JG106 | | MALE | 674 | 675 | 0.0000000 | FALSE | | 9.7320077351316 | 10.9093866489973 | 6.60233222324021 | 8.82635920474375 |
| Pat-JG108 | | MALE | 864 | 865 | 0.0000000 | FALSE | | 9.58040890602739 | 9.77840626706657 | 6.03562126310202 | 7.27333589296663 |
| Pat-JG110 | | MALE | 817 | 820 | 0.0000000 | FALSE | | 8.61189023270391 | 9.332273526541 | 5.76246523256556 | 6.61701240615369 |
| Pat-JG112 | | MALE | 942 | 946 | 0.0000000 | FALSE | 4 | 10.2396538818521 | 10.4147433658105 | 5.93257611171002 | 5.69662980238902 |
| Pat-JG114 | | MALE | 750 | 789 | 0.0000000 | FALSE | | 10.203674632942 | 9.52083719853102 | 5.7622570078745 | 5.95608432939918 |
| Pat-JG116 | | FEMALE | 681 | 684 | 0.0000000 | FALSE | 4 | 10.3308782847836 | 10.1429080142732 | 6.92340210504586 | 7.60216700800857 |
| Pat-JG118 | | MALE | 784 | 788 | 0.0000000 | FALSE | | 9.35300814449538 | 10.4372032983248 | 6.40339320827231 | 8.02719169496466 |
| Pat-JG120 | | MALE | NA | 889 | 29.0108626 | TRUE | | 9.89464424983172 | 10.0057495477186 | 6.20733397615604 | 7.59455504228822 |
| Pat-JG122 | | FEMALE | 653 | NA | NA | NA | | 10.400444512626 | 10.8863272498094 | 6.6141427762952 | 8.90157843019539 |
| Pat-JG124 | | FEMALE | 783 | 783 | 11.9920327 | FALSE | | 9.84691882827996 | 10.1535816806486 | 6.33895667917076 | 6.65695991010808 |
| Pat-JG126 | | FEMALE | 609 | 618 | 0.0000000 | TRUE | 1 | 9.08451766611881 | 9.79440513115618 | 6.21500397168323 | 7.44631815207732 |
| Pat-JG128 | | MALE | 574 | 577 | 1.7741637 | FALSE | 0 | 10.0855215322497 | 10.6369624769686 | 6.58279470387102 | 7.47742960157217 |
| Pat-JG130 | | FEMALE | 671 | 675 | 0.6899526 | FALSE | 1 | 9.79351046936302 | 10.4957846023415 | 6.4467640696073 | 7.7297283366043 |
| Pat-JG132 | REKTUM | MALE | NA | 742 | 123.8957679 | FALSE | 4 | 9.62774383385206 | 9.80422630581478 | 6.5540596662742 | 7.73240264405514 |
| Pat-JG134 | | MALE | NA | 785 | 90.9423191 | FALSE | 3a | 9.53512842427312 | 10.3664906054096 | 6.50958205723249 | 8.82234524065974 |
| Pat-JG136 | REKTUM | MALE | 587 | 590 | 120.0517464 | FALSE | 3b | 9.11731027091343 | 10.5906191986498 | 6.04509075145872 | 8.80162042491029 |
| Pat-JG138 | REKTUM | MALE | 760 | 764 | 86.3426354 | FALSE | 3b | 9.48162799167322 | 9.76861171402723 | 6.35025661090672 | 8.45695570556547 |
| Pat-JG140 | REKTUM | FEMALE | 699 | 702 | 88.0839442 | FALSE | 3a | 10.6349411023387 | 10.2721422546332 | 6.78830800893699 | 8.53024085631131 |
| Pat-JG142 | REKTUM | MALE | NA | 796 | 99.9774123 | FALSE | 4 | 9.44384991582416 | 10.3386894866311 | 6.23254699149784 | 8.69201014646551 |
| Pat-JG144 | REKTUM | MALE | 741 | 744 | 3.1540689 | FALSE | 2 | 10.1619154769658 | 10.8536160889588 | 6.54926039946853 | 7.93600945865103 |
| Pat-JG146 | REKTUM | MALE | 684 | 688 | 31.7049631 | TRUE | 2 | 9.8589728277045 | 10.2729979361636 | 6.32916743404762 | 7.93980902451441 |
| Pat-JG148 | REKTUM | FEMALE | NA | 580 | 88.9710261 | FALSE | 3a | 10.0246212337956 | 10.9485354668893 | 7.0578823432146 | 8.50487353703016 |
| Pat-JG150 | REKTUM | MALE | 850 | 854 | 79.4759646 | FALSE | 3b | 9.90872351313217 | 10.4757010296799 | 6.48673167002857 | 8.73082354339933 |
| Pat-JG152 | REKTUM | FEMALE | 852 | 854 | 85.1598595 | FALSE | 2 | 9.4557572660463 | 10.5271331527458 | 6.54919298812324 | 8.29354938049326 |
| Pat-JG154 | REKTUM | MALE | NA | 631 | 62.8842481 | TRUE | 1 | 10.2812300139627 | 10.5326419955777 | 6.7657229655633 | 7.69643954872366 |
| Pat-JG156 | REKTUM | MALE | 840 | 843 | 45.7011438 | FALSE | 1 | 10.1240905625931 | 9.70901103989512 | 6.63905013550724 | 8.10397394448937 |
| Pat-JG158 | REKTUM | FEMALE | NA | 647 | 79.5088195 | FALSE | 3b | 10.5541980136486 | 10.5802535407581 | 6.58445181925029 | 8.0926197955562 |
| Pat-JG160 | REKTUM | MALE | 791 | 794 | 86.0469414 | FALSE | 4 | 9.88905869851933 | 10.5463201580905 | 6.53017263947218 | 7.53681704366412 |
| Pat-JG162 | | MALE | 641 | 644 | 58.8759523 | TRUE | 2 | 8.96236378099289 | 9.98368260105678 | 6.02167157648896 | 6.80209756231847 |
| Pat-JG164 | REKTUM | MALE | NA | 720 | 60.0258732 | FALSE | 3a | 9.31864733650426 | 10.2421110056718 | 6.17731778417097 | 8.25465600389115 |
| Pat-JG166 | REKTUM | MALE | 815 | 818 | 61.4057784 | FALSE | 1 | 9.93984567010743 | 10.1107795486369 | 6.37137866346891 | 8.13537514450566 |
| Pat-JG168 | REKTUM | MALE | 783 | 788 | 25.3639705 | TRUE | 3b | 10.0599037676567 | 11.5651574279308 | 6.85445775520778 | 8.05038462691463 |
| Pat-JG170 | REKTUM | MALE | 952 | 956 | 71.8864864 | FALSE | 2 | 9.932600675459 | 10.5897480507154 | 6.02009257983227 | 7.70606773722108 |
| Pat-JG172 | REKTUM | FEMALE | NA | 976 | 60.7486807 | FALSE | 3a | 9.84592811485597 | 10.6059164624116 | 6.08811257866425 | 7.71221000621632 |
| Pat-JG174 | REKTUM | MALE | 793 | 796 | 71.2622436 | FALSE | 3b | 9.31891985865312 | 10.2040209364471 | 6.02508494832312 | 8.10155366708474 |
| Pat-JG176 | REKTUM | MALE | NA | 711 | 61.2086490 | FALSE | 3b | 9.35827400930955 | 9.64054058714024 | 6.09307120549181 | 7.77504602318562 |
| Pat-JG178 | REKTUM | MALE | NA | 688 | 71.9521961 | FALSE | 3b | 10.3497918589499 | 10.4210851146152 | 6.27046952026899 | 7.9349526973149 |
| Pat-JG180 | REKTUM | MALE | NA | 978 | 39.2287316 | FALSE | 3b | 9.4561269657962 | 10.4291463381227 | 5.41427943536627 | 8.07512500763003 |
| Pat-JG182 | REKTUM | MALE | NA | 748 | 34.3662088 | FALSE | 1 | 9.72093472254815 | 10.2724811946416 | 6.27909816248571 | 7.19881746886859 |
| Pat-JG184 | REKTUM | FEMALE | NA | 763 | 88.7738968 | FALSE | 2 | 10.2295043265869 | 10.3909610789031 | 6.91452686826201 | 7.49079334207042 |
| Pat-JG186 | REKTUM | FEMALE | 798 | 802 | 79.5745293 | FALSE | 4 | 10.4411512340927 | 10.6298253335457 | 6.89044455629873 | 7.5205975409638 |
| Pat-JG188 | REKTUM | FEMALE | 854 | 858 | 73.4306659 | TRUE | 2 | 9.91550729755446 | 10.1911953095551 | 6.44268656032101 | 7.85907810027254 |
| Pat-JG190 | REKTUM | MALE | 918 | 921 | 77.8003655 | FALSE | 3 | 9.9070276439687 | 10.4512905046282 | 6.64469478259682 | 8.31092175035439 |
| Pat-JG192 | REKTUM | FEMALE | 914 | 918 | 75.4348139 | FALSE | 4 | 9.70530790418866 | 9.45321793703465 | 6.49472592247793 | 8.07828547537225 |
| Pat-JG194 | REKTUM | FEMALE | 695 | 700 | 72.6750036 | FALSE | 3a | 9.96086886079383 | 9.76662157164794 | 6.66984339639354 | 7.83921768968108 |
| Pat-JG196 | REKTUM | MALE | 918 | 920 | 80.2316269 | FALSE | 1 | 10.522376601178 | 9.64270663690292 | 6.62507547401101 | 8.00690778590952 |

| | | | | | | | | | | | |
|---|---|---|---|---|---|---|---|---|---|---|---|
| Pat-JG198 | REKTUM | FEMALE | NA | 750 | 34.2676441 | TRUE | 3 | 9.32698543428459 | 10.1826968548391 | 6.20921452097672 | 7.94928840877884 |
| Pat-JG200 | REKTUM | MALE | 610 | 614 | 76.8147190 | FALSE | 2 | 10.854316245525 | 10.5825865201614 | 6.93176246016122 | 7.97786723093612 |
| Pat-JG202 | REKTUM | FEMALE | 810 | 813 | 29.7993799 | FALSE | 2 | 9.64355425827559 | 10.3262977589806 | 6.30091548435709 | 7.69047666179671 |
| Pat-JG204 | REKTUM | MALE | NA | 760 | 64.6584119 | FALSE | 3 | 9.27161022573418 | 9.0192253653749 | 5.98205165249095 | 7.95181049404982 |
| Pat-JG206 | REKTUM | FEMALE | 912 | 916 | 71.5579375 | FALSE | 3b | 10.5911242395901 | 10.90925648309 | 6.87753136441826 | 8.27603876082734 |
| Pat-JG208 | REKTUM | FEMALE | 503 | NA | NA | NA | 3b | 10.7087008159918 | 11.1696408809806 | 7.08010958566877 | 8.13883084441157 |
| Pat-JG210 | REKTUM | MALE | NA | 696 | 59.9930183 | FALSE | 1 | 10.6081459876654 | 10.2275520456921 | 7.07678302657331 | 7.95689965585809 |
| Pat-JG212 | REKTUM | MALE | NA | 789 | 60.7486807 | FALSE | 3b | 8.60010915424572 | 9.75670403301705 | 6.61681560358252 | 8.1839155427794 |
| Pat-JG214 | REKTUM | FEMALE | 714 | NA | NA | NA | 4 | 10.4535732470266 | 8.62656129494514 | 6.08242345449241 | 7.2595586564002 |
| Pat-JG216 | REKTUM | MALE | 640 | 643 | 27.1052794 | TRUE | 3b | 10.4734142702646 | 10.6156906815573 | 6.66054851991928 | 7.53261639464711 |
| Pat-JG218 | REKTUM | MALE | NA | 682 | 60.3215672 | FALSE | 4 | 9.88837265627446 | 11.0262588572588 | 6.64975033333928 | 7.7140565700071 |
| Pat-JG220 | REKTUM | MALE | 725 | 728 | 57.7917411 | FALSE | 4 | 10.4510419331833 | 10.709316241893 | 6.68715810993484 | 7.39616767850495 |
| Pat-JG222 | REKTUM | FEMALE | 767 | 768 | 47.2453233 | TRUE | 2 | 9.54905988191997 | 9.65970133328911 | 6.41800874323664 | 6.87606102333389 |
| Pat-JG224 | REKTUM | MALE | 662 | 665 | 0.0000000 | TRUE | 3a | 9.8471287269081 | 9.78624631194807 | 6.6160778132491 | 7.31022588120203 |
| Pat-JG226 | REKTUM | MALE | 596 | 600 | 12.6162755 | TRUE | 1 | 10.3267670373112 | 10.4855709199509 | 6.10002668711922 | 7.23801340671052 |
| Pat-JG228 | REKTUM | MALE | NA | 427 | 28.9122980 | TRUE | 1 | 10.0114070516876 | 10.6829733276843 | 6.5215133634938 | 7.72730684876835 |
| Pat-JG230 | REKTUM | MALE | 930 | 933 | 71.9521961 | FALSE | 2 | 10.2204830535039 | 10.6070184607782 | 6.73461231039549 | 7.2676017140131 |
| Pat-JG232 | REKTUM | MALE | 777 | 781 | 59.6973244 | FALSE | 2 | 10.0973023910621 | 10.1891679638454 | 6.81358030785512 | 8.54847802013938 |
| Pat-JG234 | REKTUM | FEMALE | 892 | 895 | 18.6287193 | TRUE | 3b | 9.78634147862271 | 10.5867883319483 | 6.67692344610538 | 7.30157435249666 |
| Pat-JG236 | REKTUM | MALE | 752 | 756 | 62.2271505 | FALSE | 3b | 9.66322096608765 | 10.5754602445943 | 6.59321193574483 | 8.09744869763277 |
| Pat-JG238 | REKTUM | MALE | 875 | 879 | 60.7158258 | FALSE | 3b | 9.20911244788602 | 10.7675865094839 | 6.5887509350207 | 7.48813852293009 |
| Pat-JG240 | REKTUM | MALE | NA | 879 | 59.4673402 | FALSE | 3b | 9.78836062691856 | 9.9111042216155 | 6.85581210810502 | 7.87520251547377 |
| Pat-JG242 | REKTUM | MALE | NA | 657 | 33.0848683 | TRUE | 3b | 10.0699016799775 | 10.3258311302058 | 6.69115331863707 | 7.8148956085 |
| Pat-JG244 | REKTUM | MALE | 733 | 738 | 2.3984065 | FALSE | 3a | 9.06641212904018 | 10.5471942769553 | 5.99384716193497 | 8.77379500725143 |
| Pat-JG246 | REKTUM | FEMALE | 755 | 758 | 14.9818271 | TRUE | 3b | 9.52941352663323 | 10.7957387019814 | 6.45191637267061 | 8.45827858869724 |
| Pat-JG248 | REKTUM | MALE | 763 | 767 | 23.0312737 | FALSE | 2 | 10.7773014270062 | 9.98611211440116 | 6.96597251245161 | 8.25813604840777 |
| Pat-JG250 | REKTUM | MALE | 901 | 905 | 12.8134048 | TRUE | 3b | 9.86203587149641 | 9.46607612471635 | 6.6849801163812 | 7.20826137185643 |
| Pat-JG252 | REKTUM | FEMALE | 815 | 819 | 47.8367112 | FALSE | 4 | 10.1049230442141 | 10.2216130623954 | 6.80886091046357 | 7.79764237108892 |
| Pat-JG254 | REKTUM | FEMALE | 609 | 613 | 18.1687509 | TRUE | 1 | 9.88418031354158 | 10.8479731559824 | 6.52667352390912 | 8.2850449225161 |
| Pat-JG256 | REKTUM | FEMALE | 552 | 555 | 48.4609540 | FALSE | 4 | 9.6165734450523 | 10.9397830135036 | 6.33841579250567 | 7.13964011856864 |
| Pat-JG258 | REKTUM | FEMALE | NA | 713 | 63.6727654 | FALSE | 3b | 9.85303713614205 | 10.2466735094762 | 6.27137977602105 | 8.37288247464671 |
| Pat-JG260 | REKTUM | FEMALE | 847 | 850 | 33.1177232 | TRUE | 3b | 9.4527026484896 | 10.2320928428814 | 6.45685834644327 | 7.1949118877766 |
| Pat-JG262 | REKTUM | MALE | 714 | 717 | 60.2230025 | FALSE | 2 | 10.1801049676744 | 11.4604779116467 | 6.48718127106043 | 7.62311603089404 |
| Pat-JG264 | REKTUM | MALE | 970 | 973 | 44.5840777 | FALSE | 2 | 10.0372728073666 | 11.5837249767879 | 6.93264777701362 | 8.32267199270536 |
| Pat-JG266 | REKTUM | FEMALE | 871 | 875 | 46.9167745 | FALSE | 3b | 10.144021596241 | 10.2424858802196 | 6.56327345495345 | 6.88393422850719 |
| Pat-JG268 | REKTUM | MALE | 846 | 850 | 59.7301793 | FALSE | 2 | 9.43147115210448 | 9.6704238255805 | 6.26479532022127 | 6.9725570318282 |
| Pat-JG270 | REKTUM | MALE | NA | 766 | 24.9697119 | TRUE | 2 | 9.69232416897864 | 10.2624985272802 | 6.40266293398973 | 7.28788081458395 |
| Pat-JG272 | REKTUM | MALE | 760 | 764 | 59.7958890 | FALSE | 3 | 9.93814493970538 | 9.90568829802604 | 6.33783447617133 | 8.25584940377644 |
| Pat-JG274 | REKTUM | MALE | 863 | 867 | 0.0000000 | TRUE | 3b | 9.85727788909805 | 10.0800843723662 | 6.4152991166405 | 7.40154128431424 |
| Pat-JG276 | REKTUM | MALE | 619 | 622 | 0.0000000 | TRUE | 1 | 9.97486422089726 | 9.98431256293956 | 6.66513384503108 | 7.2660074374623 |
| Pat-JG278 | REKTUM | FEMALE | 830 | 834 | 59.7630341 | FALSE | 4 | 9.7673333284798 | 10.9664699931108 | 6.149462534978 | 8.11600065084322 |
| Pat-JG280 | REKTUM | FEMALE | 778 | 781 | 48.4938089 | FALSE | 2 | 10.0507851454335 | 10.9313256776961 | 6.57078067999794 | 8.49407746405616 |
| Pat-JG282 | BLASE | MALE | NA | NA | NA | NA | | 9.53429623966274 | 10.132904040434 | 6.25886983805001 | 7.48101302456527 |
| Pat-JG284 | REKTUM | MALE | NA | 722 | 73.7920697 | FALSE | 4 | 10.1287058374028 | 10.7521018262584 | 6.54588164000858 | 8.25813604840777 |
| Pat-JG286 | REKTUM | MALE | 655 | 658 | 4.8296680 | TRUE | 2 | 9.76611938630267 | 9.10936828893964 | 6.25403457256147 | 6.16745096821405 |
| Pat-JG288 | REKTUM | FEMALE | 672 | 675 | 47.8038563 | FALSE | 1 | 10.0661818019134 | 10.708582228281 | 6.37226227175836 | 7.47945594012763 |
| Pat-JG290 | REKTUM | MALE | 792 | 794 | 41.1343149 | FALSE | 2 | 9.72903174602245 | 9.98249975238925 | 6.21035466608885 | 7.9277765841596 |
| Pat-JG292 | REKTUM | MALE | 955 | 958 | 47.5738722 | FALSE | 3b | 9.57870091382236 | 9.91221482158525 | 6.06953338398285 | 7.52023717068601 |
| Pat-JG294 | REKTUM | MALE | 806 | 810 | 54.6376722 | FALSE | 2 | 10.3576831527751 | 9.53409451419075 | 6.89104908355076 | 7.50268320196662 |
| Pat-JG296 | REKTUM | MALE | NA | 726 | 63.0485226 | FALSE | 1 | 9.89658767938282 | 11.3462158829689 | 6.51795872620206 | 8.66243915914903 |
| Pat-JG298 | REKTUM | FEMALE | 617 | 621 | 71.1965338 | FALSE | 2 | 9.78894788907853 | 10.6204278596121 | 6.57336439973531 | 8.64673315967772 |



| | | | | | | | | | | | |
|---|---|---|---|---|---|---|---|---|---|---|---|
| Pat-JG300 | REKTUM | MALE | NA | 689 | 0.0000000 | TRUE | 2 | 10.215784148324 | 9.99917307241859 | 6.5448179502763 | 7.2035716173109 |
| Pat-JG302 | REKTUM | MALE | 652 | 657 | 52.4035401 | FALSE | 3b | 9.7689475787815 | 8.98664623506857 | 6.56366602786653 | 7.67999196926111 |
| Pat-JG304 | REKTUM | FEMALE | 880 | 884 | 25.7910840 | TRUE | 2 | 10.7136906759326 | 10.0471488010061 | 6.58057932434449 | 8.037809983039 |
| Pat-JG306 | REKTUM | MALE | 740 | 743 | 47.8367112 | FALSE | 3 | 9.89576601791338 | 10.4659182124574 | 5.98083903680363 | 7.92704466644866 |
| Pat-JG308 | REKTUM | FEMALE | 883 | 887 | 22.9984189 | TRUE | 2 | 9.4403012244641 | 10.8415616066723 | 6.5652954813932 | 7.95138011359036 |
| Pat-JG310 | REKTUM | FEMALE | 603 | 606 | 35.8775334 | FALSE | 2 | 10.3153054148497 | 11.103676096261 | 6.45573750262466 | 7.42300963187402 |
| Pat-JG312 | REKTUM | FEMALE | NA | 923 | 54.9990760 | FALSE | 2 | 9.61985795401814 | 9.51637545681719 | 6.68618527040748 | 7.96205929295081 |
| Pat-JG314 | REKTUM | FEMALE | NA | 501 | 36.1075176 | FALSE | 3b | 10.7392351934426 | 10.6526532648296 | 7.21648310084744 | 8.88071092100141 |
| Pat-JG316 | REKTUM | MALE | NA | 876 | 33.8076757 | FALSE | 4 | 9.57859965160822 | 10.813394394519 | 6.10616749250596 | 7.60216700800857 |
| Pat-JG318 | REKTUM | MALE | 496 | 499 | 0.0000000 | TRUE | 3a | 9.63899585193679 | 10.648286056648 | 6.5689637202148 | 8.12454971510677 |
| Pat-JG320 | REKTUM | MALE | 799 | 804 | 37.2245837 | FALSE | 4 | 10.0313817874561 | 10.0651935143657 | 6.48058334129658 | 7.93147420831018 |
| Pat-JG322 | REKTUM | MALE | 738 | 743 | 55.4261894 | FALSE | 4 | 10.1002527436542 | 10.2035676789026 | 6.34342505643478 | 7.08233007862914 |
| Pat-JG324 | REKTUM | MALE | 499 | 504 | 46.4568061 | FALSE | 4 | 10.0027907637881 | 8.9194432717379 | 6.74017031334999 | 7.32621684611688 |
| Pat-JG326 | REKTUM | MALE | 819 | 824 | 36.0418078 | FALSE | 3b | 9.7139350330664 | 9.85845906892524 | 6.25673065116432 | 7.43985505965958 |
| Pat-JG328 | REKTUM | FEMALE | 708 | 712 | 0.0000000 | TRUE | 3b | 10.8512258815258 | 10.421324753968 | 7.13594328754322 | 7.41604257583028 |
| Pat-JG330 | REKTUM | MALE | 813 | 816 | 0.0000000 | TRUE | 2 | 9.16616257553876 | 9.71446410813288 | 5.43986390096236 | 7.7683117913714 |
| Pat-JG332 | REKTUM | FEMALE | 941 | 945 | 37.4545679 | FALSE | 3 | 9.63661912068068 | 9.82026591275 | 5.68509648556019 | 7.12001670454561 |
| Pat-JG334 | REKTUM | MALE | 684 | 687 | 35.8775334 | FALSE | 4 | 9.27375230382975 | 10.556789843747 | 6.15202814208141 | 7.37231050379047 |
| Pat-JG336 | REKTUM | FEMALE | 762 | 764 | 38.3745046 | FALSE | 3b | 9.72998580323565 | 11.9170575672389 | 6.28721138198921 | 7.76809472022785 |
| Pat-JG338 | REKTUM | MALE | 725 | 728 | 27.3024087 | TRUE | 3a | 9.88837265627446 | 10.6842920523487 | 6.40060310576944 | 8.67908522577772 |
| Pat-JG340 | REKTUM | MALE | 860 | 864 | 18.6287193 | TRUE | 2 | 9.92319243587035 | 9.87132422203354 | 5.2205282589978 | 6.45658047445312 |
| Pat-JG342 | REKTUM | MALE | 746 | 750 | 35.8775334 | FALSE | 3b | 9.9474572302721 | 10.1870499100759 | 6.4831773398454 | 6.47966600031446 |
| Pat-JG344 | REKTUM | FEMALE | 617 | 620 | 21.3885295 | FALSE | 3b | 10.5195752116935 | 10.2674192594574 | 7.55218340983208 | 7.43976945508425 |
| Pat-JG346 | REKTUM | FEMALE | 674 | 678 | 34.1690794 | FALSE | 2 | 10.0496002930665 | 10.2527036880592 | 6.17513316012296 | 6.77570936196489 |
| Pat-JG348 | REKTUM | MALE | 650 | NA | NA | NA | | 9.35131075560352 | 10.1635408859291 | 6.13855475409213 | 7.53023660210414 |
| Pat-JG350 | REKTUM | MALE | NA | 899 | 2.2012772 | FALSE | 2 | 10.314108587286 | 9.75274652333229 | 6.59493067652669 | 6.96336453093771 |
| Pat-JG352 | REKTUM | MALE | 736 | 738 | 23.6555165 | FALSE | 1 | 9.50418216243458 | 10.3714065116273 | 5.76552482941646 | 7.35932005743421 |
| Pat-JG354 | REKTUM | MALE | 833 | 838 | 34.1690794 | FALSE | 3b | 10.332296636157 | 10.3463498043445 | 6.36817080867901 | 7.22132675100658 |
| Pat-JG356 | REKTUM | FEMALE | 758 | 763 | 36.3375018 | FALSE | 3b | 10.0218374139692 | 11.0991228561188 | 5.81370128253054 | 8.27244205352121 |
| Pat-JG358 | REKTUM | FEMALE | 703 | 707 | 8.4437052 | TRUE | 2 | 9.868789562673 | 9.12976876175697 | 6.24878110921318 | 5.98205165249095 |
| Pat-JG360 | REKTUM | FEMALE | 634 | 637 | 36.5674860 | FALSE | 4 | 9.05069185629507 | 10.6467036009497 | 6.07223954217169 | 8.31917030340053 |
| Pat-JG362 | REKTUM | MALE | 779 | 783 | 24.9040021 | FALSE | 3a | 9.14851626384365 | 10.0916112300662 | 6.39416402700528 | 6.46858029637206 |
